# The effect of urbanization and temperature on thermal tolerance, foraging performance, and competition in cavity‐dwelling ants

**DOI:** 10.1002/ece3.10923

**Published:** 2024-02-21

**Authors:** Brooke A. Harris, Dale R. Stevens, Kaitlyn A. Mathis

**Affiliations:** ^1^ Clark University Worcester Massachusetts USA; ^2^ Bucknell University Lewisburg Pennsylvania USA

**Keywords:** cavity‐dwelling ants, climate, competition, plasticity, thermal tolerance, urbanization

## Abstract

Human disturbance including rapid urbanization and increased temperatures can have profound effects on the ecology of local populations. Eusocial insects, such as ants, have adapted to stressors of increasing temperature and urbanization; however, these evolutionary responses are not consistent among populations across geographic space. Here we asked how urbanization and incubation temperature influence critical thermal maximum (CT_max_) and various ecologically relevant behaviors in three ant species in urban and rural locations in Worcester, MA, USA. We did this by incubating colonies of three species of cavity dwelling ant (*Aphaenogaster picea*, *Tapinoma sessile*, and *Temnothorax longispinosus*) from 2 habitat types (Rural and Urban), for 60‐days at multiple temperatures. We found that incubation temperature, urbanization, and species of ant all significantly affected overall colony critical thermal maximum. We also found that recruitment time, colonization time, and defense response were significantly affected by incubation temperature and varied between species of ant, while recruitment and colonization time were additionally affected by urbanization. These variable changes in performance and competitive traits across species suggest that responses to urbanization and shifting temperatures are not universal across species. Changes in behavioral responses caused by urbanization may disrupt biodiversity, creating unusual competitive environments as a consequence of natural adaptations and cause both direct and indirect mechanisms for which human disturbance can lead to local species extinction.

## Graphical abstract

Human disturbance including rapid urbanization and increased temperatures can have profound effects on the ecology of local populations. Here, we asked how urbanization and incubation temperature influence critical thermal maximum (CT_max_) and various ecologically relevant behaviors in three ant species in urban and rural locations. We found that incubation temperature, urbanization status, and species have varying effects on ant behavior and physiology, suggesting that responses to urbanization and shifting temperatures are not universal across species or context.

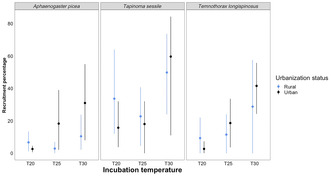

## 1 INTRODUCTION

Human disturbance in the form of shifts in climate and increasing urbanization create selective pressure on the physiology and behavior of organisms which may have cascading effects on community dynamics (McKinney, 2002, 2006; Walther, 2010). Shifts in temperature and urbanization are inextricably linked as urbanization transforms landscapes to create pockets of localized warming through loss of natural features and the addition of manmade structures that retain heat captured throughout the day (Diamond, Chick, Perez, Strickler, Martin, 2018). This ‘urban heat island’ effect can have profound impacts on organisms that thrive in and around urban environments, and in some cases can potentially act as selection pressures driving the evolution of phenotypically plastic (Diamond et al., 2017; Yilmaz et al., 2019) and non‐plastic (Martin et al., 2019) traits.

Recent studies suggest that the temperatures within urban heat islands can create evolutionary responses in organisms within urban spaces. For example, *Temnothorax curvispinosus* ants from urban environments run faster and have a higher metabolic rate than ants from rural environments, which suggests their adaptation to urban spaces (Yilmaz et al., 2019). Furthermore, urban populations of *T. curvispinosus* have evolved a higher degree of temperature‐induced plasticity in their critical thermal tolerance (CT_max_), resulting in their CT_max_ increasing when reared at higher temperatures when compared to their rural counterparts (Diamond, Chick, Perez, Strickler, Zhao, 2018). The recent expansion of urban areas creates potential environments where phenotypically plastic responses may be triggered, and selection may act upon them (Diamond, Chick, Perez, Strickler, Martin, 2018; Diamond & Martin, 2020; Levis & Pfennig, 2016).

As ectotherms, insects must adapt to changes created by urbanization to regulate their nesting and foraging behaviors and maintain an optimal body temperature (Chick et al., 2021). Foraging activity, nesting site, and type can each influence how insects are able to maintain control over colony and brood temperature. For example, to sustain proper colony temperature, ants within temperate forested ecosystems will nest underground or within acorns to ensure adequate thermal conditions within their nesting site that are distinct and separate from external environmental conditions (Bestelmeyer, 2000). Individuals can regulate their own internal temperature by foraging in optimal environmental conditions to avoid daily extremes and ultraviolet ray intensity (Chick et al., 2021; Huey et al., 2003; Markin, 1970; Muños, 2022). Furthermore, previous studies show that individuals exhibit different critical thermal maxima before and after brief extreme temperature exposure, suggesting that species‐specific thermal tolerances are also a response to local microhabitat temperature cycles (Kay & Whitford, 1978). If organisms can modulate their foraging activity to minimize exposure to higher temperatures, and exposure to these higher temperatures influences thermal maximum, they may experience different selective pressures despite living in the same environment. This variation may lead to organisms experiencing the effects of the urban heat island differently and make it difficult to make broad predictions of how organisms will adapt to one type of environmental change, and the role that phenotypic plasticity may have in the adaptation process (Levis & Pfennig, 2016; Wund, 2012). Indeed, shifting temperatures have been shown to have differing impacts on species from within the same guild and even populations of the same species (Diamond, Chick, Perez, Strickler, Martin, 2018). For example, increasing temperatures have been shown to alter nest box occupancy and colonization responses of some forest dwelling ant species in the field but not others (Diamond et al., 2016).

Ants are critical to many ecological processes within ecosystems, including the cycling of nutrients and maintenance of vegetation composition and soil structure (Del Toro et al., 2012; Sanford et al., 2009). Cavity‐dwelling ants, or ants that live in the hollowed cavities of fallen acorns, oak galls and twigs, have been shown to exhibit substantial variation in behavior in areas of increased thermal pressure. For example, the acorn nesting ant *T. curvispinosus* responds to raised temperatures with increased activity, metabolic rate, and increased energy requirements (Chick et al., 2021). These have negative fitness effects when ants are near their critical thermal maximums and can cease nesting and foraging activity altogether (Oberg et al., 2012). The variation in thermal and behavioral responses make cavity‐dwelling ants an excellent study system for analyzing these traits across species.

Here, we compare the behavioral and physiological traits of cavity‐dwelling ants (*Aphaenogastor picea*, *Tapinoma sessile*, and *Temnothorax longispinosus*) between urban and rural areas along a temperature gradient. Cavity‐dwelling ants in temperate forests are found within the second layer of leaf litter and occupy preformed plant cavities. For these ants, nest sites are ephemeral, and competition for nest sites can be strong (Herbers & Johnson, 2007). Our three focal species are all commonly found within our study sites occupying acorns. However, these species maintain differing life history strategies and compete for resources within the same ecosystems, creating larger implications for how responses to the urban heat island can influence community structure through both direct effects (changes in thermal limits) and indirect effects (behavioral modifications that influence competition). We hypothesize that each ant species will have differences in patterns of performance based on urbanization and temperature gradient, which may be due to differences in their natural histories.

*Temnothorax longispinosus* is the only obligate cavity nesting ant included within this study, meaning they require acorns, oak galls, or hollow twigs within the leaf litter as a nesting site, while our other species opportunistically nest in acorns but can be found nesting within the leaf litter itself (Helms Cahan et al., 2017). *T. longispinosus* have relatively long‐lived queens (5–15 years for this genus), and reproductive activity does not begin until their first or second year (Diamond et al., 2017; Keller, 1998). Therefore, this species has experienced few generations under recent climatic conditions, which will allow us to detect relatively rapid responses to urbanization (Diamond et al., 2017). *A. picea* is a common forest‐dwelling ant in the northeastern United States that nests within acorns and other cavities and exhibits dominant foraging behavior compared to other cavity‐dwelling ants (Helms Cahan et al., 2017). *A. picea* has previously shown the ability to acclimate to novel temperatures via heat shock response (Helms Cahan et al., 2017). Unlike *A. picea* and *T. longispinosus*, *Tapinoma sessile* are found globally within most habitats and considered urban exploiters (Buczkowski & Krushelnycky, 2012). Additionally, although native to the area of study, they share traits with invasive ant species including extreme polygyny, polydomy, unicoloniality, and ecological dominance over other native ant species, leading them to colonize many urban and temperature‐extreme habitats (Blumenfeld et al., 2022; Salyer et al., 2014).

In this study, we perform a common garden experiment to examine the impacts of urban heat island stress on the behavior and physiology of three cavity‐dwelling ant species across different habitat locations. We aimed to investigate the following questions: (1) How does critical thermal tolerance differ between cavity‐dwelling species that exist in urban and rural areas across a temperature gradient? (2) How do individual and colony‐level performance metrics vary across species and between urban and rural areas? (3) How do different temperatures impact these performance metrics? We addressed these questions by collecting ants from the field and exposing them to artificial temperature conditions in the laboratory. When studying evolutionary patterns, it is important to consider the influence of developmental plasticity (West‐Eberhard, 2003). Our study does not specifically consider developmental plasticity, and interpretations of our results here should keep this consideration in mind.

We focused our analyses on several physiological or behavioral traits that would directly link to foraging and competitive success under urban heat stressors: critical thermal maximum (CT_max_) as our physiological response and defense response, recruitment rate, and colonization rate as our behavioral responses. Examining differences in CT_max_, as it relates to temperature, species, and habitat, will allow us to observe the direct effects of urbanization and increasing temperature on these species' ability to function and forage in urbanized habitats. Comparing defense response allows us to determine how well individuals from these species can defend their territory and resources under different temperature and urbanization conditions. Recruitment rate and colonization rate are both colony‐level metrics that allow us to examine how well ants can locate and collect food resources and occupy new nest cavities. Studying these traits in tandem will allow for an analysis of competitive success and foraging ability as they are all indirect measures of fitness. Collecting across urban and rural sites will allow us to see how urbanization may affect these different performance measures, while different temperature treatments will show us how habitat variability can impact both worker and colony performance.

Rapid urbanization has occurred globally within the past century (Diamond et al., 2017; Gianotti et al., 2016). Our results will help contribute to the developing understanding of how rapid urbanization impacts species ability to perform and survive under these persisting pressures.

## 2 METHODS

### 2.1 Colony collection, identification, and maintenance

**FIGURE 1:**
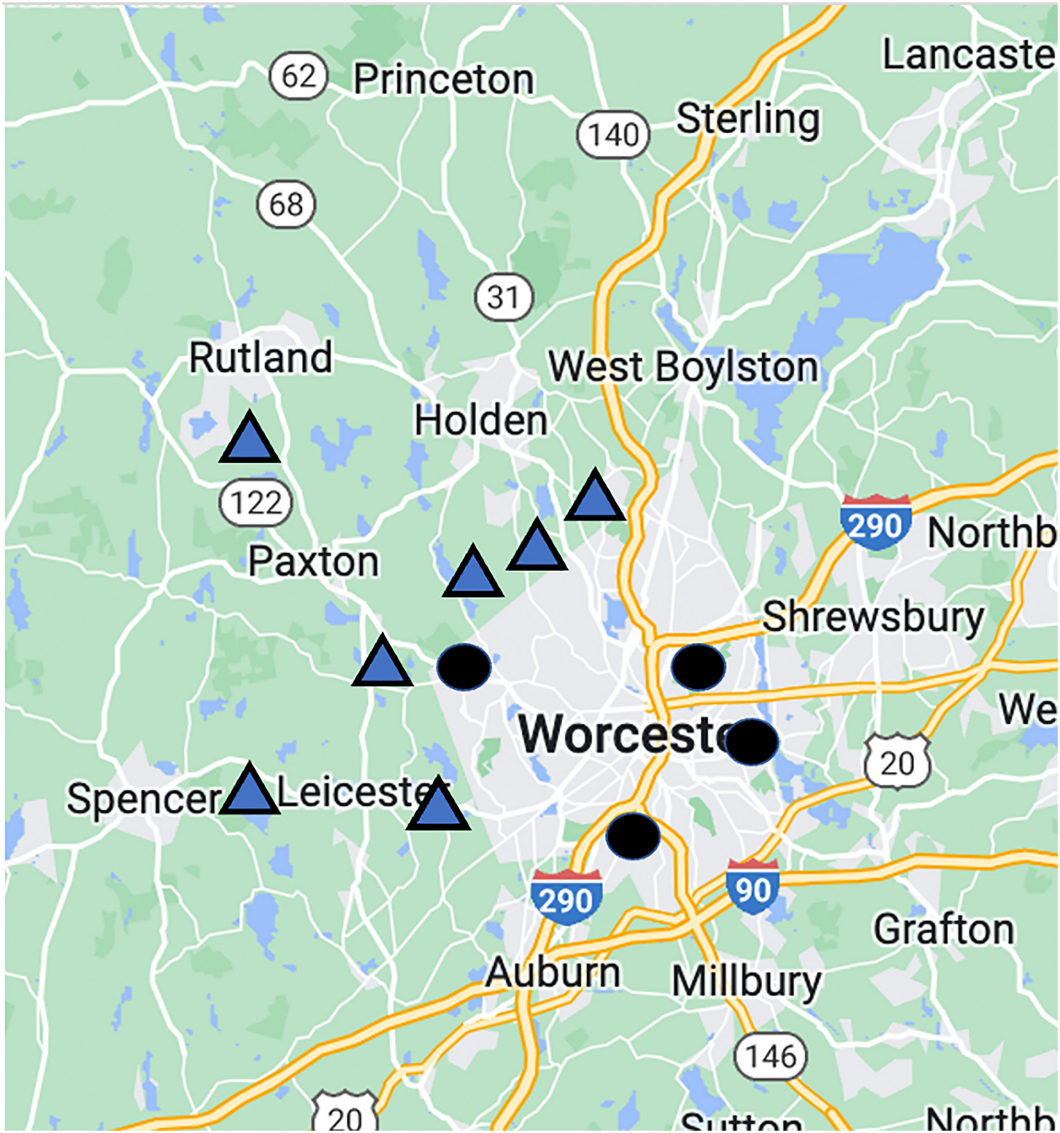
A map of rural, edge, and urban locations in Worcester County where cavity‐dwelling ants were collected. Blue triangles represent rural locations, and black circles represents urban locations.

We collected 107 cavity‐dwelling ant colonies within acorns from rural and urban areas in and around the neighboring towns of Worcester, Massachusetts, from June–August of 2021 (Figure 1). We used impervious surface area (ISA), the extent of nonevaporating impervious material that covers much of urban areas to create an urban heat island and an increase in sensible heat flux, at a 1 km buffer to define our rural (0%–5% ISA) and urban (30%–50% ISA) sites (similar methods in Yuan & Bauer, 2007) (Table S1). Our three chosen species occur in urban and rural environments across the eastern United States and inhabit single, ephemeral cavities (acorns), which allows for entire colony collection and long‐term laboratory rearing. While the precise foraging and dispersal distances for these three species are unknown, other acorn nesting ants have quite small dispersal distances, often less than 1 m (Diamond et al., 2017). We chose sites that were at minimum approximately 1–1.5 km from one another to capture colonies from different populations. We transported whole colonies in 50 mL Falcon tubes to identify and house in the lab. We then placed the ants with their original acorns in plastic artificial habitats (11 × 17 × 5 cm) coated with INSECT‐a‐SLIP (BioQuip Products Inc., Rancho Dominguez, CA) to prevent escape. We provided ants with 15 mL glass test tubes for shelter as an artificial nest, access to honey, water, and tuna (a protein source) ad libitum (similar methods in Verble‐Pearson et al., 2015).

### 2.2 Temperature treatments

We sorted colonies by species and collection location, then further assigned a treatment of one of three incubation temperatures: 20, 25, and 30°C (Table 1). We chose these temperature treatments in order to be consistent with other studies examining performance in acorn nesting ants in Cleveland, OH which has similar temperature ranges to Worcester, MA (Diamond et al., 2017). We maintained colonies within their incubation temperature treatments for 60 days in July and August 2021 to allow for ample time to acclimate (Warren et al., 2020; Yilmaz et al., 2019). Other studies with *T. curvispinosus*, another acorn nesting ant species, note that 5 weeks is a sufficient time for worker turnover (Diamond, Chick, Perez, Strickler, Zhao, 2018). All colonies used here had eggs present at the beginning of incubation and we noted worker turnover during our 60‐day incubation period, which should allow us to account for parental effects in this study. We provided colonies with a standard long‐day 14:10 L:D photoperiod beginning at 7 am to mimic natural summer light conditions. If colonies died before the end of the 60‐day incubation, we removed them from the treatment group. At the end of the incubation period, we immediately tested colonies for colony‐level performance (recruitment percentage and colonization time), individual‐level performance (defense), and CT_max_ always in that order. We then counted the number of workers within each colony including those used within assays. Colonies with fewer than 10 workers after incubation were not included in our colony‐level performance metric analysis. While some colonies did have queens (19%) at the time of collection, queen presence as a random effect did not improve the fit of our models and was therefore not included in our analyses below. No colony with a queen experienced queen mortality during the incubation period. Of the 107 originally collected colonies, 99 colonies and 626 individual ants were used in the assays described below (Table 1). These included 47 *A. picea* colonies with an average of 26.89 ± 11.65 workers, 29 *T. longispinosus* colonies with an average of 35.55 ± 18.98 workers, and 23 *T. sessile* colonies with an average of 106.52 ± 44.10 workers.

**TABLE 1:** A breakdown of the number of colonies per species that were tested from rural and urban habitats, in each incubation temperature.

Rural	T20	T25	T30	Urban	T20	T25	T30
*Aphaenogaster picea*	13	8	9	*A. picea*	5	7	5
*Temnothorax longispinosus*	3	4	4	*T. longispinosus*	6	5	7
*Tapinoma sessile*	3	4	6	*T. sessile*	4	3	3

*Note*: 99 of the original 107 colonies collected were used on performance assays.

### 2.3 Colony performance assays

We evaluated colony‐level performance through two activity assays: recruitment percentage to a food resource and nest colonization time. Recruitment percentage allows for insight into foraging speed across species, as it may take longer for some species to scout and recruit individuals under stressful environments (Bestelmeyer, 2000). To measure recruitment percentage, we first starved ants of a protein source for two weeks prior to the experiment. Then, we placed 0.1 g of tuna on the furthest edge of the artificial habitat. After 20 min, we counted the number of individuals present at the tuna to provide us with a total proportion of individuals from the colony at the food source.

To measure colonization time, we transferred all ants within a colony from their initial artificial nesting site by removing ants with soft tipped forceps and placing them into a falcon tube. The ants were then transferred to the center of an empty artificial habitat with artificial nests (empty glass test tubes and glass test tubes with water and a cotton plug) that mimic a secluded nesting site. We then recorded the colonies over a 24‐h period, and measured colonization time by the speed at which it took for a colony, including brood, to move to a new nest divided by the number of individuals within the colony (similar to methods in Charbonneau et al., 2015; Jiménez‐Soto & Philpott, 2015; Santos et al., 2005).

### 2.4 Defense assay

For each colony, we randomly selected six workers for defense assays. We first isolated workers in a foraging arena (11 × 17 × 5 cm) coated with INSECT‐a‐SLIP (BioQuip Products Inc., Rancho Dominguez, CA) for 5 min to acclimate. We then prodded individuals on their back legs with soft forceps to illicit a defense response (similar to methods in Guo et al., 2022). We recorded reactions according to the behavioral scale of 1–6 modified from Kamhi et al. (2015): (1) Nondefensive behavior, such as reversing direction or running away, (2) olfactory assessment, or antennal waving in the direction of the threat, (3) flaring mandibles at the threat, (4) adopting a threatening posture, such as an abrupt stop with flaring of mandibles, and/or raising or lowering of the gaster into a c‐posture, (5) lunging toward the threat, or (6) prolonged biting of the threat.

### 2.5 CT_max_ assays

Immediately after defense responses were recorded, we placed the same workers individually in 2 mL microcentrifuge tubes and placed the tubes within in a Stuart heating block. We then manipulated the ambient temperature beginning at 20°C and steadily increased 0.5°C every 5 min. Individuals were checked at the end of every 5 min for signs of heat exhaustion and immobility. Ants were considered immobile when they were lying on their back and their legs were no longer twitching. We recorded the temperature before that which they were declared immobile as the individual's CT_max_ (similar to methods in Cerdá et al., 1998).

### 2.6 Statistical analysis

All statistical analyses were performed with R version 4.1.2 (R Core Team, 2022). To examine how habitat type, incubation temperature, and species effect defense and CT_max_, we ran two linear mixed effects models using the lmer function in the “lme4” package (Bates et al., 2015) with defense and CT_max_ as the response variables. For each model, habitat type, incubation temperature, and species were treated as fixed factors, and colony ID was treated as a random factor. For models analyzed using the lmer function, we used the contrasts and emmeans functions in the “emmeans” package (Russell, 2018) to conduct post hoc tests for all model terms that were statistically significant. Prior to analysis, we tested the normality of our data using histograms and qqplots. Assumptions were met in all cases. We took this statistical approach because it allows us to test for evolved differences (via Urbanization), the presence of plasticity (via Temperature Treatment), evolved plasticity (via the Urbanization:Treatment interaction), and evolved plasticity between species (via Urbanization:Treatment:Species) (Ehlman et al., 2019; Levis et al., 2020; Stevens II et al., 2022; Stevens II, Graham, et al., 2023; Westrick et al., 2019; Wund et al., 2008).

To examine how recruitment percentage and colonization time were impacted by habitat type, incubation temperature, and species, we first ran two linear mixed effects models using the lmer function in the “lme4” package (Bates et al., 2015) with recruitment percentage and colonization time as the response variables. For each model, habitat type, incubation temperature, and species were treated as fixed factors, and colony size was treated as a random factor. However, for both models, the random effect of colony size was not significant (recruitment percentage: *p* = 1, colonization time: *p* = .98), so we chose to remove the random effect from our models. We then ran models using the anova function in base R with recruitment percentage or colonization time as the response variable and habitat type, incubation temperature and species as fixed factors. For these models, we used the TukeyHSD function in base R, to run post hoc analyses. Prior to analysis, we tested the normality of our data using histograms and qqplots. Assumptions were met in all cases.

We ran Mantel tests using the mantel.test function in the “ape” package (Paradis & Schliep, 2019) if the distance between sites influenced our our CT_max_, defense, colonization, and recruitment assay results for each species across sites (Anastasio et al., 2021; Guillot & Rousset, 2013). Furthermore, because we used the same ants in the defense assays and CT_max_ assays, we wanted to determine whether ants that responded the most defensively were not also ants with the lowest CT_max_ results due to stress or energy expenditure. To test this, we ran a linear regression model to ask if the results from our defense assay predicted our CT_max_ results. Lastly, all figures were created using the ggplot function in the ggplot2 package (Wickham, 2016).

## 3 RESULTS

### 3.1 Recruitment percentage

**FIGURE 2:**
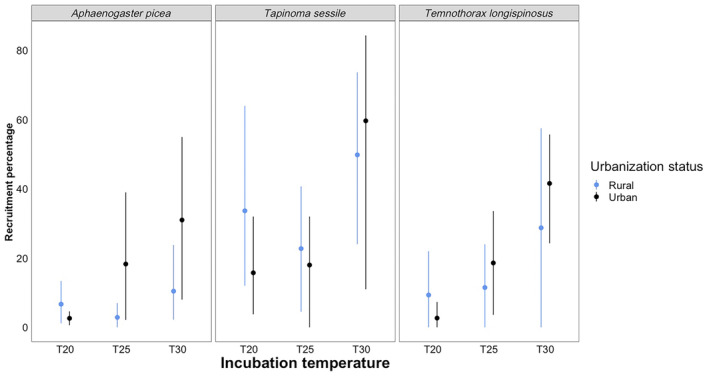
Results for our recruitment percentage tests across three different species at different temperature intervals. From left to right, the boxes display results for *Aphaenogastor picea*, *Tapinoma*, and *Temnothorax longispinosus*. Blue represents ants from rural populations, and black represents ants from urban populations. The *x*‐axis is our three different incubation temperatures (20, 25, and 30°C), and the *y*‐axis is recruitment percentage, measured as the number of individuals present divided by the number of individuals in the colony total. Error bars represent 95% confidence intervals.

We found a species difference in recruitment percentages for ant species (*F*_2,81_ = 10.0067, *p* = .0001, Table 2, Figure 2). In particular, *T. sessile* recruited significantly more workers to baits than *A. picea* (*p* < .0001, Table S2) and *T. longispinosus* (*p* = .0492, Table S2). Incubation also showed significant differences in our Recruitment Percentage model as well (*F*_2,81_ = 11.2030, *p* = <0.0001). Ants incubated in the 30° treatment had significantly higher recruitment percentage than those from both 25° (*p* = .0002) and 20° treatments (*p* < .0001, Table S3).

**TABLE 2:** Results for our linear mixed effects model on the recruitment percentage response variable.

Model formula		Sums Sq	df	*F*	*p*
**Recruitment Percentage**~Urbanization:Species:Incubation Temperature	**Species**	**8662**	**2**	**10.0067**	**.0001**
	**Urbanization**	458	1	1.0586	.3066
	Incubation	**9697**	**2**	**11.203**	**.0001**
	Species:Urbanization	808	2	0.9335	.3974
	Species:Incubation	2281	4	1.3176	.2705
	Urbanization:Incubation	2248	2	2.5969	.0807
	Species:Urbanization:Incubation	88	4	0.0507	.9951
	Residual	35,057	81		

*Note*: Statistically significant results are bolded in the analysis. Bolded values indicate statistical significance.

### 3.2 Colonization time

**FIGURE 3:**
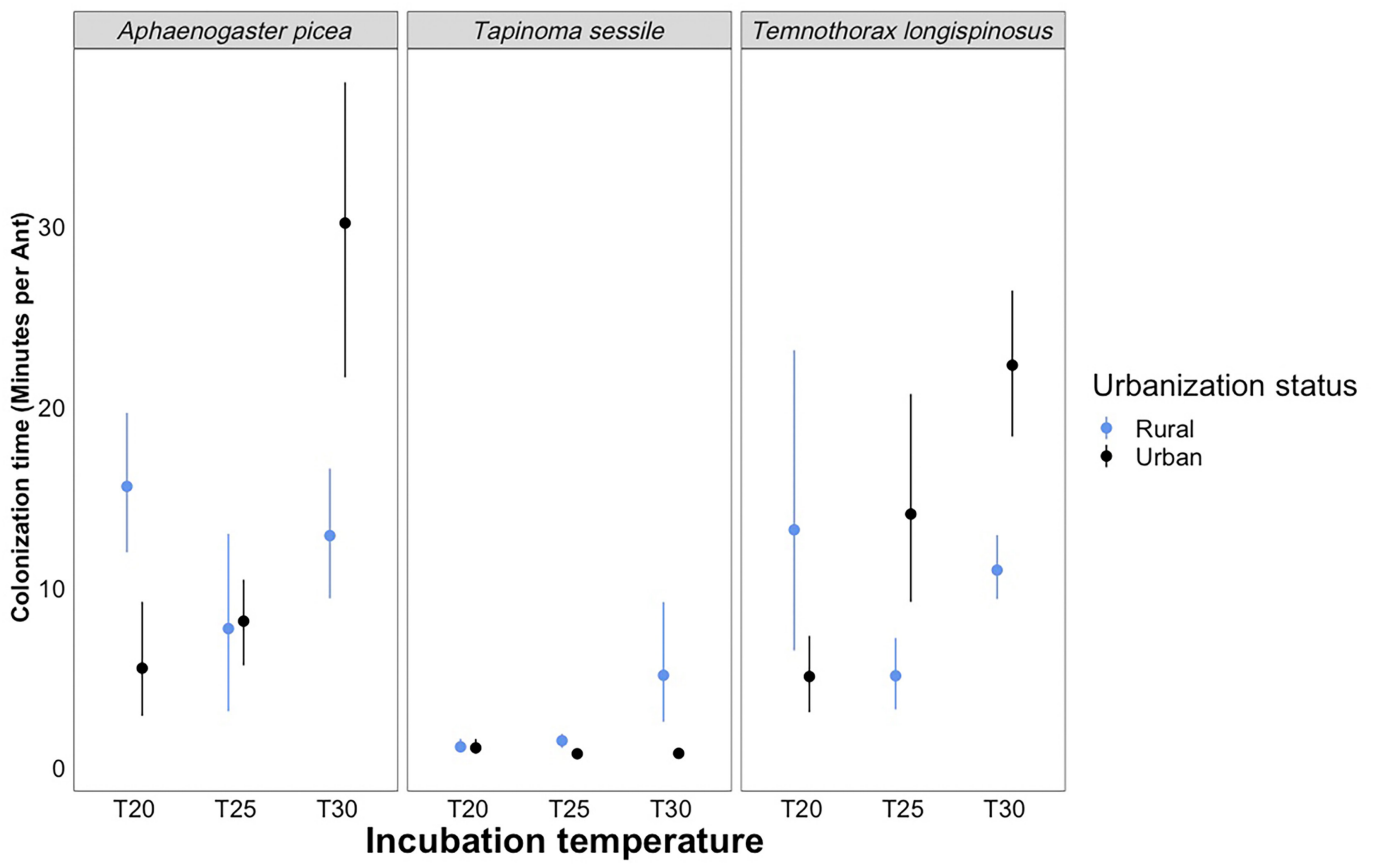
Results for our colonization time tests across three different species at different temperature intervals. From left to right, the boxes display results for *Aphaenogastor picea*, *Tapinoma sessile*, and *Temnothorax longispinosus*. Blue represents ants from rural populations, and black represents ants from urban populations. The *x*‐axis is our three different incubation temperatures (20, 25, and 30°C), and the *y*‐axis is colonization time measured in minutes.

The individual terms of species (*F*_2,81_ = 30.1862, *p* < .0001, Table 3) and incubation (*F*_2,81_ = 18.3825, *p* < .0001, Table 3) were statistically significant in our model for colonization time (Table 3, Figure 3). *T. sessile* had significantly a faster colonization time than both *T. longispinosus* and *A. picea* (*p* < .0001, Table S4). Furthermore, differences between colonization time in the temperature treatments were driven by 30° incubation treatments having slower colonization times than 20° (*p* < .0001) and 25° (*p* < .0001) treatments (Table S5).

**TABLE 3:** Results for our linear mixed effects model on the colonization time response variable.

Model formula	Sums Sq	df	*F*	*p*	
**Colonization Time**~Urbanization:Species:Incubation Temperature	**Species**	**2027.23**	**2**	**30.1862**	**<.0001**
	Urbanization	47.88	1	1.426	.2359
	**Incubation**	**1234.52**	**2**	**18.3825**	**<.0001**
	Species:Urbanization	101.09	2	1.5053	.2281
	**Species:Incubation**	**378.31**	**4**	**2.8166**	
	**Urbanization:Incubation**	**1087.94**	**2**	**16.1999**	**<.0001**
	**Species:Urbanization:Incubation**	**703.76**	**4**	**5.2396**	**.0008**
	Residual	2719.88	81		

*Note*: Statistically significant results are bolded in the analysis. Bolded values indicate statistical significance.

**FIGURE 4:**
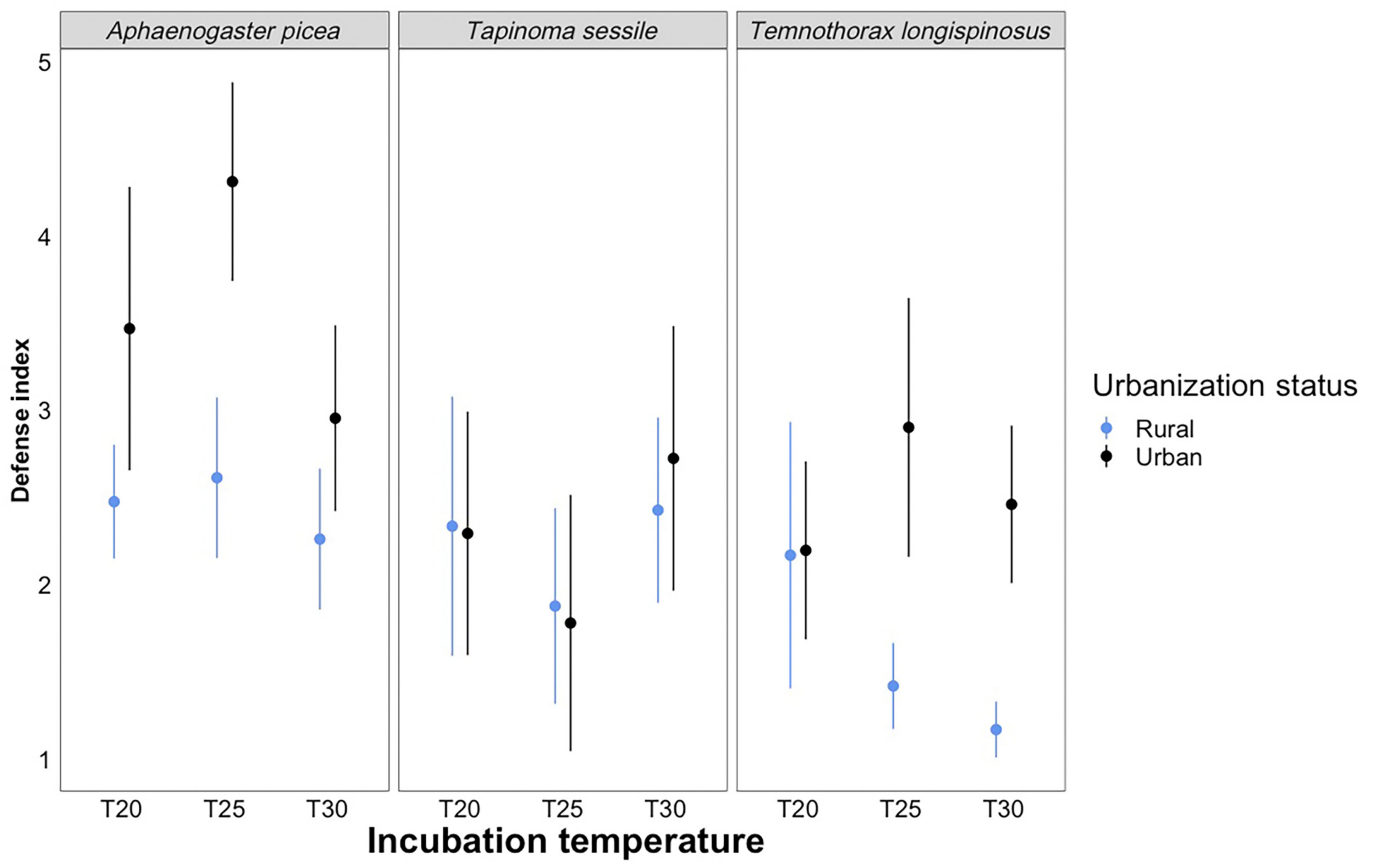
Results for our defense response tests across three different species at different temperature intervals. From left to right, the boxes display results for *Aphaenogastor picea*, *Tapinoma sessile*, and *Temnothorax longispinosus*. Blue represents ants from rural populations, and black represents ants from urban populations. The *x*‐axis is our three different incubation temperatures (20, 25, and 30°C), and the *y*‐axis represents the following values on the defense index scale: (1) Nondefensive behavior, such as reversing direction or running away, (2) olfactory assessment, or antennal waving in the direction of the threat, (3) flaring mandibles at the threat, (4) adopting a threatening posture, defined as an abrupt stop, flaring of mandibles, and raising of the gaster, (5) lunging toward the threat, or (6) prolonged biting of the threat.

Three of our interaction terms were statistically significant as well (Table 3, Figure 4). We saw an effect of Species:Incubation (*F*_4,81_ = 2.8166, *p* = .0304, Table S6) and Urbanization:Incubation (*F*_2,81_ = 16.1999, *p* < .0001, Table S7). Finally, there is a significant three‐way interaction between species, urbanization, and incubation (*F*_4,81_ = 5.2396, *p* = .0008, Table 3), with each species displaying unique responses (Figure 4), and most of these responses being significantly different from each other (Table S8).

### 3.3 Defense index

We found differences in defense based on both species (*F*_2,99_ = 14.6143, *p* < .0001) and urbanization (*F*_1,99_ = 16.5716, *p* < .0001, Table 4). *A. picea* had significantly higher defensiveness than both *T. sessile* and *T. longispinosus* (*p* < .0028, Table S9), and urban ants were significantly more defensive than ants from rural populations (*p* = .0003, Table S10). Urban colonies of *A. picea* demonstrated the most defensive behavior, while rural *T. longispinosus* colonies demonstrated the least defensive behavior (Figure 3). Overall, *A. picea* colonies exhibit the most defensive behavior across all incubation temperatures and collection locations (Figure 3). Furthermore, we found two significant interactions in our model, Species:Urbanization (*F*_2,99_ = 3.4855, *p* = .0344) and Species:Incubation Temperature (*F*_4,99_ = 2.7817, *p* = .0307). Urban *A. picea* was the most defensive species in the experiment (*p* < .0050, Table S11). *A. picea* in the 25°C treatment was the most defensive (*p* < .0380), while *T. longispinosus* in the 30°C treatment was the least defensive (*p* < .0380, Table S12).

**TABLE 4:** Results for our linear mixed effects model on the defensive assay response variable.

Model formula	Random effects			Fixed effects				
		Variance	*p*		Sums Sq	df	*F*	*p*
**Defensive Assay**~Urbanization:Species:Incubation Temperature + (1|Colony ID)	**Colony ID**	**0.268**	**.0002**	**Species**	**63.993**	**2**	**14.614**	**<.0001**
	Residual	2.189		**Urbanization**	**36.282**	**1**	**16.572**	**<.0001**
				Incubation	1.889	2	0.432	.6507
				**Species:Urbanization**	**15.263**	**2**	**3.486**	**.0344**
				**Species:Incubation**	**24.361**	**4**	**2.782**	**.0307**
				Urbanization:Incubation	6.943	2	1.586	.2100
				Species:Urbanization:Incubation	11.394	4	1.301	.2749

*Note*: Colony ID was included as a random effect, while all other terms are coded as a fixed effect. Statistically significant results are bolded in the analysis.

### 3.4 CT_max_

**FIGURE 5:**
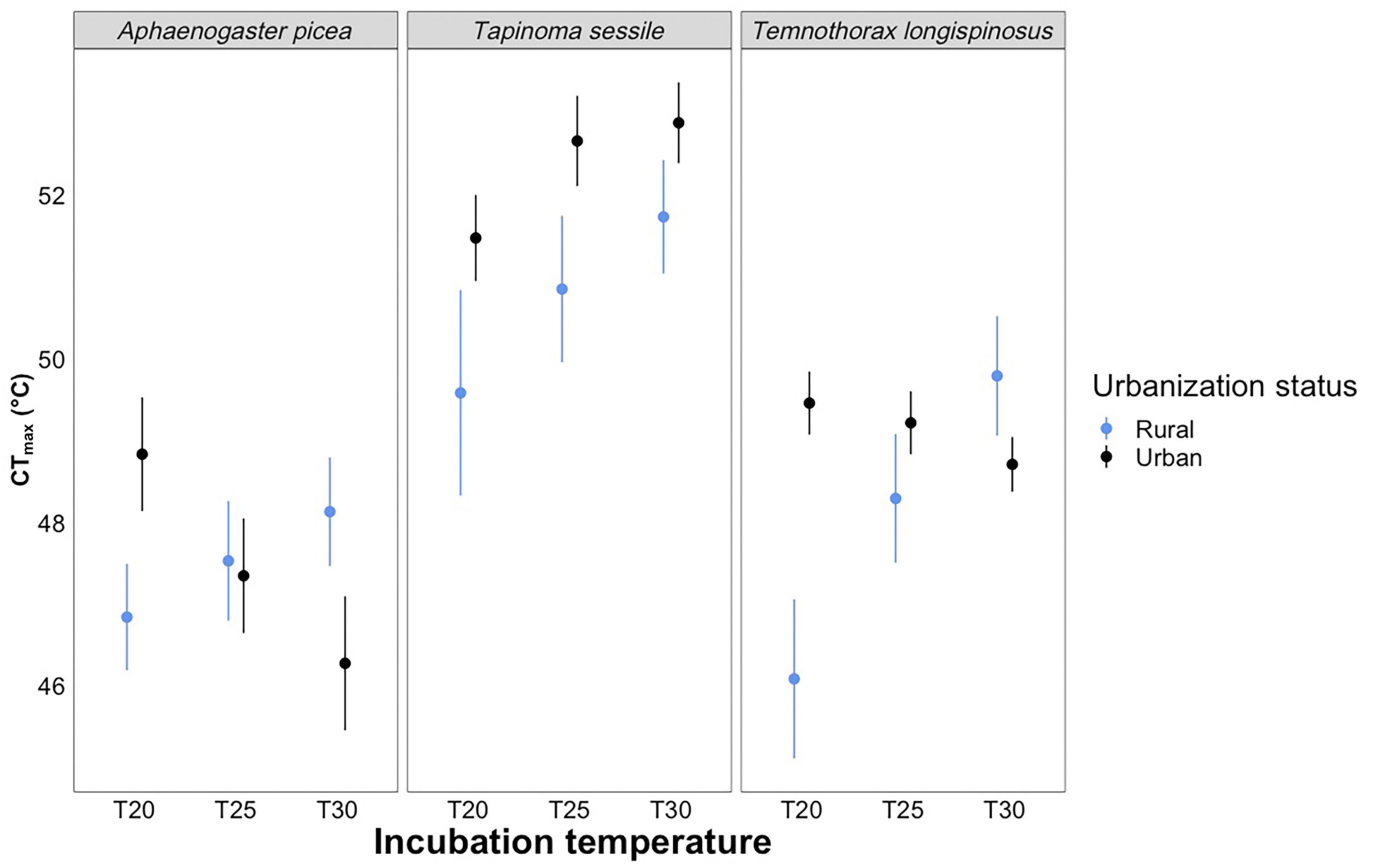
Results for our critical thermal maximum tests across three different species at different temperature intervals. From left to right, the boxes display results for *Aphaenogastor picea*, *Tapinoma sessile*, and *Temnothorax longispinosus*. Blue represents ants from rural populations, and black represents ants from urban populations. The *x*‐axis is our three different incubation temperatures (20, 25, and 30°C), and the *y*‐axis is temperature in degrees Celsius.

Our CT_max_ model had 6 significant factors (Table 5). Species was significant (*F*_2,106_ = 101.5489, *p* < .0001) with all species being statistically different from each other (*p* < .0005, Table S13). Overall, *T. sessile* has a significantly higher CT_max_, followed by *T. longispinosus* and *A. picea* (Figure 5). Urbanization was also significant (*F*_1,106_ = 14.5098, *p* = .0002), with ants from urban habitats having a higher CT_max_ overall than rural ants (Table S14). Incubation temperature also influenced CT_max_, being explained by differences between the 20 and 30°C treatments (Table S15). The highest CT_max_ is in *T. sessile* colonies collected at urban sites in the median temperature treatment, while the lowest CT_max_ is in *T. longispinosus* collected at rural locations at the lowest temperature treatment (Figure 5). Furthermore, the interactions terms of Species:Urbanization (*F*_2,106_ = 4.7668, *p* = .0104), Species:Incubation (*F*_4,106_ = 4.3854, *p* = .0025) and Urbanization: Incubation were significant (*F*_2,106_ = 14.1637, *p* < .0001). All interactions had numerous statistically significant differences appear in the post hoc analyses (Tables S16–S18). Notably, Rural *T. sessile* and *A. picea* were the groups that differed the most from other groups in the Species:Urbanization effect (Table S16). *Tapinoma sessile* had the highest CT_max_ at 30°C when compared to any other species at any other temperature treatment (Figure 5); however, the significant differences in the post hoc tests were primarily driven by all three species at different temperatures (Table S17). Finally, rural populations at 20°C had significantly lower CT_max_ than all other urbanization:incubation combinations (Table S18).

**TABLE 5:** Results for our linear mixed effects model on the CT_max_ response variable.

Model formula	Random effects			Fixed effects				
		Variance	*p*		Sums Sq	df	*F*	*p*
**CT**_**max**_~Urbanization:Species:Incubation Temperature + (1|Colony ID)	**Colony ID**	**0.5462**	**<.0001**	**Species**	**790.06**	**2**	**101.549**	**<.0001**
	Residual	3.89		**Urbanization**	**56.44**	**1**	**14.510**	**.0002**
				**Incubation**	**38.08**	**2**	**4.895**	**.0093**
				**Species:Urbanization**	**37.09**	**2**	**4.767**	**.0104**
				**Species:Incubation**	**68.24**	**4**	**4.385**	**.0025**
				**Urbanization:Incubation**	**110.97**	**2**	**14.264**	**<.0001**
				Species:Urbanization:Incubation	27.27	4	1.752	.1439

*Note*: Colony ID was included as a random effect, while all other terms are coded as a fixed effect. Statistically significant results are bolded in the analysis.

The results of our mantel tests demonstrated that distance between sites did not explain the variation in each phenotype for all species (Table S19). Although we found a weak positive relationship between defense and CT_max_ (*F*_1,624_ = 4.722, *R* ^2^ = .006, *p* = .0302), this suggests that the stress or energy expenditure during the defense assays did not cause ants to have a lower CT_max_, but instead might indicate that there is some correlation between higher CT_max_ and higher defense response.

## 4 DISCUSSION

The goal of our study was to compare the behavior and physiology of cavity‐dwelling ant species across developmental temperatures and urbanization. We also aimed to determine if ants that exist in urban areas have evolved behavioral and physiological adaptations to urbanization and to what degree complex patterns of phenotypic plasticity have evolved. Overall, we found that species differed in their thermal tolerance and behaviors and the patterns of their responses varied based on both urbanization and temperature. Below we discuss each of the phenotypes we examined in depth.

Recruitment to a food source is a key metric of performance as competitive ability is highly dependent upon ability to successfully scout for and collect food, especially in areas of urbanization where resources are scarce for certain species (Diamond et al., 2012, but see also Penick et al., 2015). In our experiment, recruitment percentage was significantly predicted by the main effects of species and incubation temperature (Table 2). *Tapinoma sessile* appears to have the higher recruitment percentage among the three species and considerably less variation among habitat types and temperatures (Figure 2). This may be one of the contributing factors that has led to *T. sessile* being labeled an “Urban Exploiter” (McKinney, 2002, 2006), as it can recruit colony members to locations faster than other species. Furthermore, it appears that, on average, urban ant populations also display the higher recruitment percentages, relative to rural ant populations. This may be particularly important to urban populations of ants as recent work shows ants in urban spaces that discover food resources tend to be the ones that monopolize them, a reversal on the more common “dominance‐discovery” trade‐off (Dáttilo & MacGregor‐Fors, 2021).

Cavity‐dwelling ants occupy ephemeral, limited habitats. Thus, fast colonization time should be especially advantageous with already restricted material and may allow ants to outcompete other species (Yilmaz et al., 2019). We found that colonization time was significantly influenced by every factor and interaction in our analysis except urbanization and the interaction between urbanization and species. Overall, *T. sessile* demonstrates the fastest colonization time regardless of incubation temperature and urbanization (Figure 3). As previously stated, *T. sessile* share many traits with invasive species which allow them to successfully occupy urbanized areas, including faster colonization speeds which increase the ability to translocate the colony in times of need or stress (Buczkowski & Krushelnycky, 2012). Furthermore, *A. picea* displayed the most variable responses across habitat type and temperature, with rural populations slowing down at the colder temperature treatment and urban populations slowing down at the warmer temperature treatment (Figure 3). *Temnothorax longispinosus* showed its own unique response to temperature. Urban *T. longispinosus* ants showed slower colonization times as incubation temperature increased, whereas rural *T. longispinosus* ants did not have significantly different colonization times based on incubation temperature. These are, interestingly, different trends than have been previously reported for this species, which show *T. longispinosus* increasing its colonization and occupancy of nest boxes at forested field sites with increasing temperatures (Diamond et al., 2016). Furthermore, it is interesting to note that the significant 3‐way interaction in our model for colonization time suggests that each species possesses its own unique degree of thermal plasticity based on the habitat in which it was sampled (Figure 1). More specifically, it appears that this is driven by Urban *A. picea* at 30°C differing from Rural *A. picea* at 20°C suggesting that urban environments are selecting on phenotypes expressed at the higher temperatures we tested, but only in *A. picea* (Table S1).

Our defense index response suggests that *A. picea* is the most defensive species in our experiment, averaged across habitats and temperatures (Figure 4). Furthermore, ants from urban populations appear to be more defensive on average than those from rural habitats (Figure 4). Thus, it appears that urban environments may select for an overall increase in defense response across ant species. Given the competitive nature that urban environments may present to organisms, this increased defense response may be needed to compete with other ants in these environments. However, it is worth noting that intra and interspecific competition strategies for ants may differ given some invasive urban exploiting ant populations show low genetic differentiation and a loss of intraspecific defense response which ultimately allows them to live in higher densities (Blumenfeld et al., 2022). Lastly, our defense index suggests that for urban populations of two species (*A. picea* and *T. longispinosus*) at 25°C produce the most defensive behaviors (Figure 4). This may be because 25°C is close to an optimum temperature for these species in a competitive setting and is not influenced by cold or heat stress.

Critical thermal maximum differed across species with *T. sessile* having the highest CT_max_ across all collection locations and incubation temperatures (Table 5, Figure 5), suggesting this species is better able to function at higher temperatures. *Temnothorax longispinosus* had on average the second highest CT_max_, followed by *A. picea* (Figure 2, Table S13). This result is consistent with other studies that show *T. longispinosus* has a relatively high upper thermal tolerance when compared to other community members (Diamond et al., 2016). However, our results differ from another study that reports a higher mean CT_max_ for *T. longispinosus* than *T. sessile* (Bujan et al., 2020). These differences may be due to acclimation of the ants in our study to lab temperatures or population‐level differences in the ants collected here compared with other studies. Furthermore, we found that urban ant populations, regardless of species, tend to have a higher CT_max_ than rural ant populations (Table 4, Figure 5). Species also demonstrate evolved differences in CT_max_ among habitat types as indicated by the significant species:urbanization interaction term in our model (Table 5). Indeed, *T. sessile* and *T. longispinous* appear to have evolved generally higher CT_max_ in urban habitats, regardless of temperature. However, urban and rural ants in *A. picea* appear to show opposite trends along the manipulated temperatures, which resulted in their overall average CT_max_ being much lower than *T. longispinosus* (Figure 5, Table S13). Finally, our model produced significant species:urbanization and urbanization:incubation interaction terms, suggesting that there are evolved patterns of plasticity in ant CT_max_ across species and habitat types, but the lack of a species:urbanization:incubation interaction suggests that there are still some similarities between species and habitat types in the plasticity of CT_max_. Rural populations across all three species do show the same degree of plasticity in thermal tolerance, as their CT_max_ increases with increasing incubation temperature. This is likely the result of thermal acclimation which can encompass a diverse array of metabolic, physiological and behavioral responses including changes in membrane lipid composition (e.g. Hazel, 1979; Overgaard et al., 2008), expression of heat‐shock proteins (e.g. Colinet et al., 2013; Tomanek & Somero, 1999), or behavioral changes (e.g. Lagerspetz, 2006) and can ultimately involve changes to the transcriptome, proteome, and metabolome of an organism (e.g. Collier et al., 2006; Kristensen et al., 2016; MacMillan et al., 2016). This trend of increasing CT_max_ with increasing incubation temperature is observed in the literature and is repeatable among many organisms (Angilletta, 2009). The complexity appears to be in how urban populations have evolved their plasticity, as there is not a consistent trend among the three species to suggest that there is one adaptive pattern of plasticity that may be evolving in urban environments, nor the statistical evidence in our post‐hoc tests to point to one clear trend. Interestingly, the pattern of producing a higher thermal tolerance at higher temperatures is not seen in two of our 3 species (Figure 5), suggesting that while higher temperatures can indeed prepare organisms for higher temperatures, some organisms lack that capacity to produce an adaptive plastic response to higher temperatures.

Our results suggest that individual species respond to stress brought on by urbanization uniquely, and we cannot assume uniform reactions across all ants. Our study highlights the importance of considering species differences when making broader conclusions about ant behavior and abilities. This is especially pertinent as even populations of the same species sometimes do not display the same evolutionary responses to rapid environmental change (Diamond, Chick, Perez, Strickler, & Martin, 2018), and sometimes complex experimental manipulations are needed to observe differences between urban and rural populations (de Tranaltes et al., 2022). This is likely because species possess multidimensional plasticity (Westneat et al., 2019), and it may be that in order to determine the true expression patters of plasticity, multiple variables need to be manipulated for observers to see species‐ or population‐specific responses (Stevens II, Wund, & Mathis, 2023).

Species demonstrating, or not demonstrating, complex patterns of phenotypic plasticity can make drawing general conclusions difficult and future studies should continue to explore how the changes in performance due to rising temperatures may more broadly impact insect communities as a whole, and how altered ant behaviors may cause cascading effects on the surrounding communities and ecosystems. This is especially important as the population dynamics of invasive species (and species more broadly) can be influenced by environmental factors (see Pierce, 2012 for an example in fish, Northern Pike). Thus, a scenario may exist where studying complex patterns of phenotypic expression are the only way to determine community‐level effects as each community may be experiencing its own unique environment, and thus the species within are expressing their own unique patters of trait expression.

Our study focuses on cavity‐dwelling ants, and similar studies that examine ants that occupy different niches may produce differing results. Additionally, more studies on how increasing temperatures alter natural life history cycles of organisms intimately tied to ants may provide more understanding of how the ecosystem will respond to climate change and urbanization. For example, it has been shown that increased climate warming or climate variability can cause a dramatic increase of stress on oak trees, altering acorn production (decreasing size and quantity), seedling survival and reproductive timing (Askeyev et al., 2005). Changing temperatures are also causing shifts in reproductive timing of oaks, causing acorns to be produced and dropped much earlier in the season than previously recorded which could cause conflicting life history cycles (Askeyev et al., 2005). Additionally, generalist acorn borers that hollow acorns prior to ant colonization have shown an inability to consistently adjust oviposition adequately to increasing stochastic events influenced by climate change, resulting in potential loss of a reproductive season for species that rely on these habitats (Bonal et al., 2010). These slow but permanent alterations of natural life history cycles threaten the ways in which ants (especially cavity dwelling ants) interact with their environment and should be analyzed for a thorough understanding of the risk of increased temperatures.

While temperature rise and urban sprawl continues, studying the ways in which cavity‐dwelling ants adapt to these stressors can provide insight into the future of biodiversity and the ways in which conservation efforts should be applied to these areas of change. Here, we provide the first study to address these metrics across multiple ant species from different habitats, providing a greater understanding into how intraspecific competition may respond to human development. Furthermore, we highlight that adaptation, either through plasticity or selection on standing genetic variation may be complex and make general trends hard to make. Our study reaffirms the important phenotypic plasticity in response to thermal tolerance, but also may explain why trends across insect thermal limits suggest that plasticity is “weak but pervasive” (Weaving et al., 2020). Our results also support the idea of evolved differences in these same tolerance traits (Diamond et al., 2017; Martin et al., 2019). Furthermore, we also emphasize how these changes in traits may alter the ability of certain species to outcompete others, and how this may alter ant biodiversity in areas of immense thermal pressure induced by urbanization.

## AUTHOR CONTRIBUTIONS

**Brooke A. Harris:** Conceptualization (equal); data curation (lead); investigation (lead); methodology (equal); writing – original draft (equal). **Dale R. Stevens II:** Conceptualization (supporting); formal analysis (equal); methodology (supporting); visualization (equal); writing – original draft (supporting); writing – review and editing (supporting). **Kaitlyn A. Mathis:** Conceptualization (equal); data curation (supporting); formal analysis (equal); funding acquisition (lead); investigation (supporting); methodology (equal); resources (lead); visualization (equal); writing – original draft (equal); writing – review and editing (lead).

## FUNDING INFORMATION

This work was funded through Clark University.

## CONFLICT OF INTEREST STATEMENT

The authors have no conflicts of interest.

## PERMISSION TO REPRODUCE MATERIAL FROM OTHER SOURCES

There is no material reproduced from other sources in this work.

## Supporting information


Table S1.

## Data Availability

The data that support the findings of this study is available on dryad (https://datadryad.org/stash/share/CKfGS4tZI7cpq992mjwn5pQpYLcBBVDgQ‐wyiOwN57w).
