# Supplementary material for: The effect of urbanization and temperature on thermal tolerance, foraging performance, and competition in cavity‐dwelling ants
Source: Ecol Evol. 2024 Feb 21;14(2):e10923. doi: 10.1002/ece3.10923 (PMC10880040; doi:10.1002/ece3.10923)
Supplement: Supplementary file 1 — Table S1. [file ECE3-14-e10923-s001.docx]

| **Supplementary Table 1:** Impervious surface area % for each of our study sites | |
| --- | --- |
| Collection location | ISA (%) |
| BZ | 0 |
| CW | 35 |
| GHP | 43 |
| KW | 2 |
| LP | 46 |
| MM | 0 |
| NW | 0 |
| RSP | 0 |
| SF | 0 |
| SF | 30 |
| USP | 0 |

| **Supplementary Table 2:** Post-hoc tests examining the differences between Species in their Recruitment Rates | | | | |
| --- | --- | --- | --- | --- |
| Contrast | diff | lwr | upr | p adj |
| **Tapinoma sessile-Aphaenogaster picea** | **23.5791** | **10.9395** | **36.2187** | **0.0001** |
| Temnothorax longispinosus-Aphaenogaster picea | 9.6720 | -2.0568 | 21.4009 | 0.1265 |
| **Temnothorax longispinosus-Tapinoma sessile** | **-13.9070** | **-27.7757** | **-0.0384** | **0.0492** |

| **Supplementary Table 3:** Post-hoc tests examining the differences between Incubation Temperatures in their Recruitment Rates | | | | |
| --- | --- | --- | --- | --- |
| Contrast | diff | lwr | upr | p adj |
| T25-T20 | 4.1025 | -8.2323 | 16.4373 | 0.7077 |
| **T30-T20** | **22.3953** | **10.3485** | **34.4421** | **0.0001** |
| **T30-T25** | **18.2928** | **5.9580** | **30.6276** | **0.0019** |

| **Supplementary Table 4:** Post-hoc tests examining the differences between Species in their Colonization Rate | | | | |
| --- | --- | --- | --- | --- |
| Contrast | diff | lwr | upr | p adj |
| **Tapinoma sessile-Aphaenogaster picea** | **-10.9475** | **-14.4682** | **-7.4269** | **<0.0001** |
| Temnothorax longispinosus-Aphaenogaster picea | -0.6638 | -3.9307 | 2.6032 | 0.8786 |
| **Temnothorax longispinosus-Tapinoma sessile** | **10.2837** | **6.4208** | **14.1467** | **<0.0001** |

| **Supplementary Table 5:** Post-hoc tests examining the differences between Temperatures in their Colonization Rate | | | | |
| --- | --- | --- | --- | --- |
| Contrast | diff | lwr | upr | p adj |
| T25-T20 | -1.8549 | -5.2906 | 1.5808 | 0.4055 |
| **T30-T20** | **6.3501** | **2.9946** | **9.7056** | **0.0001** |
| **T30-T25** | **8.2050** | **4.7693** | **11.6407** | **<0.0001** |

| **Supplementary Table 6:** Post-hoc tests examining the differences in Colonization Rate between different combinations of Species and Incubation Temperature | | | | |
| --- | --- | --- | --- | --- |
| Contrast | diff | lwr | upr | p adj |
| **Tapinoma sessile:T20-Aphaenogaster picea:T20** | **-11.5724** | **-19.7991** | **-3.3457** | **0.0008** |
| Temnothorax longispinosus:T20-Aphaenogaster picea:T20 | -5.3298 | -12.8697 | 2.2101 | 0.3827 |
| Aphaenogaster picea:T25-Aphaenogaster picea:T20 | -5.1335 | -11.5903 | 1.3233 | 0.2317 |
| **Tapinoma sessile:T25-Aphaenogaster picea:T20** | **-11.7439** | **-19.9706** | **-3.5172** | **0.0006** |
| Temnothorax longispinosus:T25-Aphaenogaster picea:T20 | -2.5752 | -10.1151 | 4.9648 | 0.9743 |
| Aphaenogaster picea:T30-Aphaenogaster picea:T20 | 6.2932 | -0.2881 | 12.8746 | 0.0721 |
| **Tapinoma sessile:T30-Aphaenogaster picea:T20** | **-9.2384** | **-16.7783** | **-1.6985** | **0.0058** |
| Temnothorax longispinosus:T30-Aphaenogaster picea:T20 | 5.3405 | -1.7277 | 12.4087 | 0.2939 |
| Temnothorax longispinosus:T20-Tapinoma sessile:T20 | 6.2426 | -3.0649 | 15.5501 | 0.4558 |
| Aphaenogaster picea:T25-Tapinoma sessile:T20 | 6.4389 | -2.0150 | 14.8929 | 0.2837 |
| Tapinoma sessile:T25-Tapinoma sessile:T20 | -0.1715 | -10.0435 | 9.7006 | 1.0000 |
| Temnothorax longispinosus:T25-Tapinoma sessile:T20 | 8.9972 | -0.3102 | 18.3047 | 0.0662 |
| **Aphaenogaster picea:T30-Tapinoma sessile:T20** | **17.8657** | **9.3162** | **26.4151** | **<0.0001** |
| Tapinoma sessile:T30-Tapinoma sessile:T20 | 2.3340 | -6.9735 | 11.6415 | 0.9966 |
| **Temnothorax longispinosus:T30-Tapinoma sessile:T20** | **16.9129** | **7.9833** | **25.8426** | **<0.0001** |
| Aphaenogaster picea:T25-Temnothorax longispinosus:T20 | 0.1963 | -7.5909 | 7.9835 | 1.0000 |
| Tapinoma sessile:T25-Temnothorax longispinosus:T20 | -6.4141 | -15.7216 | 2.8934 | 0.4179 |
| Temnothorax longispinosus:T25-Temnothorax longispinosus:T20 | 2.7546 | -5.9517 | 11.4610 | 0.9840 |
| **Aphaenogaster picea:T30-Temnothorax longispinosus:T20** | **11.6230** | **3.7322** | **19.5138** | **0.0004** |
| Tapinoma sessile:T30-Temnothorax longispinosus:T20 | -3.9086 | -12.6150 | 4.7977 | 0.8825 |
| **Temnothorax longispinosus:T30-Temnothorax longispinosus:T20** | **10.6703** | **2.3691** | **18.9715** | **0.0030** |
| Tapinoma sessile:T25-Aphaenogaster picea:T25 | -6.6104 | -15.0643 | 1.8435 | 0.2513 |
| Temnothorax longispinosus:T25-Aphaenogaster picea:T25 | 2.5583 | -5.2289 | 10.3455 | 0.9798 |
| **Aphaenogaster picea:T30-Aphaenogaster picea:T25** | **11.4267** | **4.5634** | **18.2900** | **<0.0001** |
| Tapinoma sessile:T30-Aphaenogaster picea:T25 | -4.1049 | -11.8921 | 3.6823 | 0.7566 |
| **Temnothorax longispinosus:T30-Aphaenogaster picea:T25** | **10.4740** | **3.1426** | **17.8054** | **0.0006** |
| Temnothorax longispinosus:T25-Tapinoma sessile:T25 | 9.1687 | -0.1388 | 18.4762 | 0.0568 |
| Aphaenogaster picea:T30-Tapinoma sessile:T25 | 18.0371 | 9.4877 | 26.5866 | 0.0000 |
| Tapinoma sessile:T30-Tapinoma sessile:T25 | 2.5055 | -6.8020 | 11.8130 | 0.9945 |
| **Temnothorax longispinosus:T30-Tapinoma sessile:T25** | **17.0844** | **8.1548** | **26.0140** | **<0.0001** |
| **Aphaenogaster picea:T30-Temnothorax longispinosus:T25** | **8.8684** | **0.9776** | **16.7592** | **0.0161** |
| Tapinoma sessile:T30-Temnothorax longispinosus:T25 | -6.6632 | -15.3696 | 2.0431 | 0.2776 |
| Temnothorax longispinosus:T30-Temnothorax longispinosus:T25 | 7.9157 | -0.3855 | 16.2169 | 0.0737 |
| **Tapinoma sessile:T30-Aphaenogaster picea:T30** | **-15.5316** | **-23.4224** | **-7.6408** | **<0.0001** |
| Temnothorax longispinosus:T30-Aphaenogaster picea:T30 | -0.9527 | -8.3941 | 6.4886 | 1.0000 |
| **Temnothorax longispinosus:T30-Tapinoma sessile:T30** | **14.5789** | **6.2778** | **22.8801** | **<0.0001** |

| **Supplementary Table 7:** Post-hoc tests examining the differences in Colonization Rate between different combinations of Urbanization Status and Incubation Temperature | | | | |
| --- | --- | --- | --- | --- |
| Contrast | diff | lwr | upr | p adj |
| **Urban:T20-Rural:T20** | **-6.5035** | **-12.3459** | **-0.6611** | **0.0202** |
| **Rural:T25-Rural:T20** | **-5.8211** | **-11.5605** | **-0.0816** | **0.0449** |
| Urban:T25-Rural:T20 | -3.4515 | -9.2939 | 2.3909 | 0.5199 |
| Rural:T30-Rural:T20 | -0.4475 | -5.9354 | 5.0405 | 0.9999 |
| **Urban:T30-Rural:T20** | **8.3934** | **2.5510** | **14.2358** | **0.0010** |
| Rural:T25-Urban:T20 | 0.6824 | -5.3969 | 6.7616 | 0.9995 |
| Urban:T25-Urban:T20 | 3.0520 | -3.1246 | 9.2285 | 0.7012 |
| **Rural:T30-Urban:T20** | **6.0560** | **0.2136** | **11.8984** | **0.0377** |
| **Urban:T30-Urban:T20** | **14.8969** | **8.7204** | **21.0734** | **<0.0001** |
| Urban:T25-Rural:T25 | 2.3696 | -3.7097 | 8.4488 | 0.8641 |
| Rural:T30-Rural:T25 | 5.3736 | -0.3659 | 11.1131 | 0.0797 |
| **Urban:T30-Rural:T25** | **14.2145** | **8.1353** | **20.2938** | **<0.0001** |
| Rural:T30-Urban:T25 | 3.0040 | -2.8384 | 8.8464 | 0.6647 |
| **Urban:T30-Urban:T25** | **11.8449** | **5.6684** | **18.0215** | **<0.0001** |
| **Urban:T30-Rural:T30** | **8.8409** | **2.9985** | **14.6833** | **0.0004** |

| **Supplementary Table 8:** Post-hoc tests examining the differences in Colonization Rate between different combinations of Species, Urbanization Status, and Incubation Temperature | | | | |
| --- | --- | --- | --- | --- |
| Contrast | diff | lwr | upr | p adj |
| **Tapinoma sessile:Rural:T20-Aphaenogaster picea:Rural:T20** | **-14.4268** | **-27.8029** | **-1.0507** | **0.0216** |
| Temnothorax longispinosus:Rural:T20-Aphaenogaster picea:Rural:T20 | -2.4022 | -15.7784 | 10.9739 | 1.0000 |
| Aphaenogaster picea:Urban:T20-Aphaenogaster picea:Rural:T20 | -10.0689 | -21.0585 | 0.9207 | 0.1120 |
| **Tapinoma sessile:Urban:T20-Aphaenogaster picea:Rural:T20** | **-14.4834** | **-26.4239** | **-2.5428** | **0.0044** |
| **Temnothorax longispinosus:Urban:T20-Aphaenogaster picea:Rural:T20** | **-10.5330** | **-20.8400** | **-0.2260** | **0.0398** |
| Aphaenogaster picea:Rural:T25-Aphaenogaster picea:Rural:T20 | -7.8761 | -17.2602 | 1.5081 | 0.2135 |
| **Tapinoma sessile:Rural:T25-Aphaenogaster picea:Rural:T20** | **-14.0854** | **-26.0260** | **-2.1448** | **0.0066** |
| Temnothorax longispinosus:Rural:T25-Aphaenogaster picea:Rural:T20 | -10.4904 | -22.4310 | 1.4501 | 0.1555 |
| Aphaenogaster picea:Urban:T25-Aphaenogaster picea:Rural:T20 | -7.4627 | -17.2530 | 2.3277 | 0.3667 |
| **Tapinoma sessile:Urban:T25-Aphaenogaster picea:Rural:T20** | **-14.8093** | **-28.1855** | **-1.4332** | **0.0156** |
| Temnothorax longispinosus:Urban:T25-Aphaenogaster picea:Rural:T20 | -1.5307 | -12.5203 | 9.4589 | 1.0000 |
| Aphaenogaster picea:Rural:T30-Aphaenogaster picea:Rural:T20 | -2.7254 | -11.7811 | 6.3303 | 0.9997 |
| **Tapinoma sessile:Rural:T30-Aphaenogaster picea:Rural:T20** | **-10.4629** | **-20.7699** | **-0.1559** | **0.0428** |
| Temnothorax longispinosus:Rural:T30-Aphaenogaster picea:Rural:T20 | -4.6370 | -16.5776 | 7.3036 | 0.9944 |
| **Aphaenogaster picea:Urban:T30-Aphaenogaster picea:Rural:T20** | **14.5919** | **3.6023** | **25.5815** | **0.0010** |
| **Tapinoma sessile:Urban:T30-Aphaenogaster picea:Rural:T20** | **-14.7887** | **-28.1648** | **-1.4126** | **0.0159** |
| Temnothorax longispinosus:Urban:T30-Aphaenogaster picea:Rural:T20 | 6.7182 | -3.0721 | 16.5085 | 0.5569 |
| Temnothorax longispinosus:Rural:T20-Tapinoma sessile:Rural:T20 | 12.0246 | -5.0267 | 29.0758 | 0.5074 |
| Aphaenogaster picea:Urban:T20-Tapinoma sessile:Rural:T20 | 4.3579 | -10.8932 | 19.6091 | 0.9999 |
| Tapinoma sessile:Urban:T20-Tapinoma sessile:Rural:T20 | -0.0565 | -16.0066 | 15.8935 | 1.0000 |
| Temnothorax longispinosus:Urban:T20-Tapinoma sessile:Rural:T20 | 3.8938 | -10.8730 | 18.6607 | 1.0000 |
| Aphaenogaster picea:Rural:T25-Tapinoma sessile:Rural:T20 | 6.5507 | -7.5874 | 20.6889 | 0.9669 |
| Tapinoma sessile:Rural:T25-Tapinoma sessile:Rural:T20 | 0.3414 | -15.6086 | 16.2914 | 1.0000 |
| Temnothorax longispinosus:Rural:T25-Tapinoma sessile:Rural:T20 | 3.9364 | -12.0136 | 19.8864 | 1.0000 |
| Aphaenogaster picea:Urban:T25-Tapinoma sessile:Rural:T20 | 6.9642 | -7.4468 | 21.3751 | 0.9520 |
| Tapinoma sessile:Urban:T25-Tapinoma sessile:Rural:T20 | -0.3825 | -17.4338 | 16.6688 | 1.0000 |
| Temnothorax longispinosus:Urban:T25-Tapinoma sessile:Rural:T20 | 12.8961 | -2.3550 | 28.1473 | 0.2033 |
| Aphaenogaster picea:Rural:T30-Tapinoma sessile:Rural:T20 | 11.7014 | -2.2209 | 25.6237 | 0.2116 |
| Tapinoma sessile:Rural:T30-Tapinoma sessile:Rural:T20 | 3.9639 | -10.8030 | 18.7307 | 0.9999 |
| Temnothorax longispinosus:Rural:T30-Tapinoma sessile:Rural:T20 | 9.7898 | -6.1602 | 25.7398 | 0.7399 |
| **Aphaenogaster picea:Urban:T30-Tapinoma sessile:Rural:T20** | **29.0187** | **13.7676** | **44.2698** | **0.0000** |
| Tapinoma sessile:Urban:T30-Tapinoma sessile:Rural:T20 | -0.3619 | -17.4132 | 16.6894 | 1.0000 |
| **Temnothorax longispinosus:Urban:T30-Tapinoma sessile:Rural:T20** | **21.1450** | **6.7341** | **35.5560** | **0.0001** |
| Aphaenogaster picea:Urban:T20-Temnothorax longispinosus:Rural:T20 | -7.6666 | -22.9178 | 7.5845 | 0.9333 |
| Tapinoma sessile:Urban:T20-Temnothorax longispinosus:Rural:T20 | -12.0811 | -28.0311 | 3.8689 | 0.3779 |
| Temnothorax longispinosus:Urban:T20-Temnothorax longispinosus:Rural:T20 | -8.1308 | -22.8976 | 6.6361 | 0.8677 |
| Aphaenogaster picea:Rural:T25-Temnothorax longispinosus:Rural:T20 | -5.4738 | -19.6120 | 8.6643 | 0.9946 |
| Tapinoma sessile:Rural:T25-Temnothorax longispinosus:Rural:T20 | -11.6832 | -27.6332 | 4.2668 | 0.4381 |
| Temnothorax longispinosus:Rural:T25-Temnothorax longispinosus:Rural:T20 | -8.0882 | -24.0382 | 7.8618 | 0.9285 |
| Aphaenogaster picea:Urban:T25-Temnothorax longispinosus:Rural:T20 | -5.0604 | -19.4714 | 9.3505 | 0.9982 |
| Tapinoma sessile:Urban:T25-Temnothorax longispinosus:Rural:T20 | -12.4071 | -29.4584 | 4.6442 | 0.4502 |
| Temnothorax longispinosus:Urban:T25-Temnothorax longispinosus:Rural:T20 | 0.8716 | -14.3796 | 16.1227 | 1.0000 |
| Aphaenogaster picea:Rural:T30-Temnothorax longispinosus:Rural:T20 | -0.3232 | -14.2455 | 13.5991 | 1.0000 |
| Tapinoma sessile:Rural:T30-Temnothorax longispinosus:Rural:T20 | -8.0607 | -22.8275 | 6.7061 | 0.8755 |
| Temnothorax longispinosus:Rural:T30-Temnothorax longispinosus:Rural:T20 | -2.2348 | -18.1848 | 13.7152 | 1.0000 |
| **Aphaenogaster picea:Urban:T30-Temnothorax longispinosus:Rural:T20** | **16.9941** | **1.7430** | **32.2453** | **0.0144** |
| Tapinoma sessile:Urban:T30-Temnothorax longispinosus:Rural:T20 | -12.3865 | -29.4377 | 4.6648 | 0.4532 |
| Temnothorax longispinosus:Urban:T30-Temnothorax longispinosus:Rural:T20 | 9.1204 | -5.2905 | 23.5314 | 0.6939 |
| Tapinoma sessile:Urban:T20-Aphaenogaster picea:Urban:T20 | -4.4145 | -18.4235 | 9.5946 | 0.9995 |
| Temnothorax longispinosus:Urban:T20-Aphaenogaster picea:Urban:T20 | -0.4641 | -13.1097 | 12.1814 | 1.0000 |
| Aphaenogaster picea:Rural:T25-Aphaenogaster picea:Urban:T20 | 2.1928 | -9.7126 | 14.0982 | 1.0000 |
| Tapinoma sessile:Rural:T25-Aphaenogaster picea:Urban:T20 | -4.0165 | -18.0256 | 9.9925 | 0.9999 |
| Temnothorax longispinosus:Rural:T25-Aphaenogaster picea:Urban:T20 | -0.4216 | -14.4306 | 13.5875 | 1.0000 |
| Aphaenogaster picea:Urban:T25-Aphaenogaster picea:Urban:T20 | 2.6062 | -9.6219 | 14.8343 | 1.0000 |
| Tapinoma sessile:Urban:T25-Aphaenogaster picea:Urban:T20 | -4.7405 | -19.9916 | 10.5107 | 0.9996 |
| Temnothorax longispinosus:Urban:T25-Aphaenogaster picea:Urban:T20 | 8.5382 | -4.6697 | 21.7460 | 0.6600 |
| Aphaenogaster picea:Rural:T30-Aphaenogaster picea:Urban:T20 | 7.3435 | -4.3048 | 18.9917 | 0.7000 |
| Tapinoma sessile:Rural:T30-Aphaenogaster picea:Urban:T20 | -0.3941 | -13.0396 | 12.2515 | 1.0000 |
| Temnothorax longispinosus:Rural:T30-Aphaenogaster picea:Urban:T20 | 5.4319 | -8.5772 | 19.4409 | 0.9945 |
| **Aphaenogaster picea:Urban:T30-Aphaenogaster picea:Urban:T20** | **24.6608** | **11.4529** | **37.8686** | **<0.0001** |
| Tapinoma sessile:Urban:T30-Aphaenogaster picea:Urban:T20 | -4.7198 | -19.9710 | 10.5313 | 0.9996 |
| **Temnothorax longispinosus:Urban:T30-Aphaenogaster picea:Urban:T20** | **16.7871** | **4.5590** | **29.0152** | **0.0005** |
| Temnothorax longispinosus:Urban:T20-Tapinoma sessile:Urban:T20 | 3.9504 | -9.5298 | 17.4306 | 0.9998 |
| Aphaenogaster picea:Rural:T25-Tapinoma sessile:Urban:T20 | 6.6073 | -6.1812 | 19.3957 | 0.9172 |
| Tapinoma sessile:Rural:T25-Tapinoma sessile:Urban:T20 | 0.3980 | -14.3689 | 15.1648 | 1.0000 |
| Temnothorax longispinosus:Rural:T25-Tapinoma sessile:Urban:T20 | 3.9929 | -10.7739 | 18.7598 | 0.9999 |
| Aphaenogaster picea:Urban:T25-Tapinoma sessile:Urban:T20 | 7.0207 | -6.0687 | 20.1101 | 0.8902 |
| Tapinoma sessile:Urban:T25-Tapinoma sessile:Urban:T20 | -0.3260 | -16.2760 | 15.6240 | 1.0000 |
| Temnothorax longispinosus:Urban:T25-Tapinoma sessile:Urban:T20 | 12.9527 | -1.0564 | 26.9617 | 0.1038 |
| Aphaenogaster picea:Rural:T30-Tapinoma sessile:Urban:T20 | 11.7579 | -0.7915 | 24.3073 | 0.0926 |
| Tapinoma sessile:Rural:T30-Tapinoma sessile:Urban:T20 | 4.0204 | -9.4598 | 17.5006 | 0.9998 |
| Temnothorax longispinosus:Rural:T30-Tapinoma sessile:Urban:T20 | 9.8464 | -4.9205 | 24.6132 | 0.6076 |
| **Aphaenogaster picea:Urban:T30-Tapinoma sessile:Urban:T20** | **29.0753** | **15.0662** | **43.0843** | **<0.0001** |
| Tapinoma sessile:Urban:T30-Tapinoma sessile:Urban:T20 | -0.3054 | -16.2554 | 15.6447 | 1.0000 |
| **Temnothorax longispinosus:Urban:T30-Tapinoma sessile:Urban:T20** | **21.2016** | **8.1122** | **34.2910** | **<0.0001** |
| Aphaenogaster picea:Rural:T25-Temnothorax longispinosus:Urban:T20 | 2.6569 | -8.6214 | 13.9353 | 1.0000 |
| Tapinoma sessile:Rural:T25-Temnothorax longispinosus:Urban:T20 | -3.5524 | -17.0326 | 9.9278 | 1.0000 |
| Temnothorax longispinosus:Rural:T25-Temnothorax longispinosus:Urban:T20 | 0.0426 | -13.4377 | 13.5228 | 1.0000 |
| Aphaenogaster picea:Urban:T25-Temnothorax longispinosus:Urban:T20 | 3.0703 | -8.5482 | 14.6888 | 1.0000 |
| Tapinoma sessile:Urban:T25-Temnothorax longispinosus:Urban:T20 | -4.2763 | -19.0432 | 10.4905 | 0.9998 |
| Temnothorax longispinosus:Urban:T25-Temnothorax longispinosus:Urban:T20 | 9.0023 | -3.6433 | 21.6479 | 0.4902 |
| Aphaenogaster picea:Rural:T30-Temnothorax longispinosus:Urban:T20 | 7.8076 | -3.1990 | 18.8141 | 0.4967 |
| Tapinoma sessile:Rural:T30-Temnothorax longispinosus:Urban:T20 | 0.0701 | -11.9870 | 12.1271 | 1.0000 |
| Temnothorax longispinosus:Rural:T30-Temnothorax longispinosus:Urban:T20 | 5.8960 | -7.5842 | 19.3762 | 0.9808 |
| **Aphaenogaster picea:Urban:T30-Temnothorax longispinosus:Urban:T20** | **25.1249** | **12.4793** | **37.7704** | **<0.0001** |
| Tapinoma sessile:Urban:T30-Temnothorax longispinosus:Urban:T20 | -4.2557 | -19.0226 | 10.5111 | 0.9999 |
| **Temnothorax longispinosus:Urban:T30-Temnothorax longispinosus:Urban:T20** | **17.2512** | **5.6327** | **28.8697** | **0.0001** |
| Tapinoma sessile:Rural:T25-Aphaenogaster picea:Rural:T25 | -6.2093 | -18.9978 | 6.5791 | 0.9500 |
| Temnothorax longispinosus:Rural:T25-Aphaenogaster picea:Rural:T25 | -2.6143 | -15.4028 | 10.1741 | 1.0000 |
| Aphaenogaster picea:Urban:T25-Aphaenogaster picea:Rural:T25 | 0.4134 | -10.3948 | 11.2216 | 1.0000 |
| Tapinoma sessile:Urban:T25-Aphaenogaster picea:Rural:T25 | -6.9333 | -21.0714 | 7.2049 | 0.9457 |
| Temnothorax longispinosus:Urban:T25-Aphaenogaster picea:Rural:T25 | 6.3454 | -5.5600 | 18.2508 | 0.8952 |
| Aphaenogaster picea:Rural:T30-Aphaenogaster picea:Rural:T25 | 5.1507 | -4.9969 | 15.2982 | 0.9279 |
| Tapinoma sessile:Rural:T30-Aphaenogaster picea:Rural:T25 | -2.5869 | -13.8652 | 8.6915 | 1.0000 |
| Temnothorax longispinosus:Rural:T30-Aphaenogaster picea:Rural:T25 | 3.2391 | -9.5494 | 16.0275 | 1.0000 |
| **Aphaenogaster picea:Urban:T30-Aphaenogaster picea:Rural:T25** | **22.4680** | **10.5626** | **34.3734** | **<0.0001** |
| Tapinoma sessile:Urban:T30-Aphaenogaster picea:Rural:T25 | -6.9126 | -21.0508 | 7.2255 | 0.9470 |
| **Temnothorax longispinosus:Urban:T30-Aphaenogaster picea:Rural:T25** | **14.5943** | **3.7861** | **25.4025** | **0.0007** |
| Temnothorax longispinosus:Rural:T25-Tapinoma sessile:Rural:T25 | 3.5950 | -11.1719 | 18.3618 | 1.0000 |
| Aphaenogaster picea:Urban:T25-Tapinoma sessile:Rural:T25 | 6.6227 | -6.4667 | 19.7122 | 0.9298 |
| Tapinoma sessile:Urban:T25-Tapinoma sessile:Rural:T25 | -0.7239 | -16.6739 | 15.2261 | 1.0000 |
| Temnothorax longispinosus:Urban:T25-Tapinoma sessile:Rural:T25 | 12.5547 | -1.4543 | 26.5638 | 0.1338 |
| Aphaenogaster picea:Rural:T30-Tapinoma sessile:Rural:T25 | 11.3600 | -1.1894 | 23.9094 | 0.1236 |
| Tapinoma sessile:Rural:T30-Tapinoma sessile:Rural:T25 | 3.6225 | -9.8577 | 17.1027 | 0.9999 |
| Temnothorax longispinosus:Rural:T30-Tapinoma sessile:Rural:T25 | 9.4484 | -5.3184 | 24.2152 | 0.6766 |
| **Aphaenogaster picea:Urban:T30-Tapinoma sessile:Rural:T25** | **28.6773** | **14.6683** | **42.6863** | **<0.0001** |
| Tapinoma sessile:Urban:T30-Tapinoma sessile:Rural:T25 | -0.7033 | -16.6533 | 15.2467 | 1.0000 |
| **Temnothorax longispinosus:Urban:T30-Tapinoma sessile:Rural:T25** | **20.8036** | **7.7142** | **33.8930** | **<0.0001** |
| Aphaenogaster picea:Urban:T25-Temnothorax longispinosus:Rural:T25 | 3.0278 | -10.0616 | 16.1172 | 1.0000 |
| Tapinoma sessile:Urban:T25-Temnothorax longispinosus:Rural:T25 | -4.3189 | -20.2689 | 11.6311 | 0.9999 |
| Temnothorax longispinosus:Urban:T25-Temnothorax longispinosus:Rural:T25 | 8.9597 | -5.0493 | 22.9688 | 0.6773 |
| Aphaenogaster picea:Rural:T30-Temnothorax longispinosus:Rural:T25 | 7.7650 | -4.7844 | 20.3144 | 0.7282 |
| Tapinoma sessile:Rural:T30-Temnothorax longispinosus:Rural:T25 | 0.0275 | -13.4527 | 13.5077 | 1.0000 |
| Temnothorax longispinosus:Rural:T30-Temnothorax longispinosus:Rural:T25 | 5.8534 | -8.9134 | 20.6203 | 0.9930 |
| **Aphaenogaster picea:Urban:T30-Temnothorax longispinosus:Rural:T25** | **25.0823** | **11.0733** | **39.0914** | **<0.0001** |
| Tapinoma sessile:Urban:T30-Temnothorax longispinosus:Rural:T25 | -4.2983 | -20.2483 | 11.6517 | 0.9999 |
| **Temnothorax longispinosus:Urban:T30-Temnothorax longispinosus:Rural:T25** | **17.2086** | **4.1192** | **30.2980** | **0.0012** |
| Tapinoma sessile:Urban:T25-Aphaenogaster picea:Urban:T25 | -7.3467 | -21.7576 | 7.0643 | 0.9254 |
| Temnothorax longispinosus:Urban:T25-Aphaenogaster picea:Urban:T25 | 5.9320 | -6.2961 | 18.1601 | 0.9504 |
| Aphaenogaster picea:Rural:T30-Aphaenogaster picea:Urban:T25 | 4.7372 | -5.7870 | 15.2615 | 0.9747 |
| Tapinoma sessile:Rural:T30-Aphaenogaster picea:Urban:T25 | -3.0003 | -14.6188 | 8.6182 | 1.0000 |
| Temnothorax longispinosus:Rural:T30-Aphaenogaster picea:Urban:T25 | 2.8257 | -10.2637 | 15.9151 | 1.0000 |
| **Aphaenogaster picea:Urban:T30-Aphaenogaster picea:Urban:T25** | **22.0546** | **9.8264** | **34.2827** | **<0.0001** |
| Tapinoma sessile:Urban:T30-Aphaenogaster picea:Urban:T25 | -7.3261 | -21.7370 | 7.0849 | 0.9270 |
| **Temnothorax longispinosus:Urban:T30-Aphaenogaster picea:Urban:T25** | **14.1809** | **3.0182** | **25.3435** | **0.0021** |
| Temnothorax longispinosus:Urban:T25-Tapinoma sessile:Urban:T25 | 13.2787 | -1.9725 | 28.5298 | 0.1661 |
| Aphaenogaster picea:Rural:T30-Tapinoma sessile:Urban:T25 | 12.0839 | -1.8384 | 26.0062 | 0.1699 |
| Tapinoma sessile:Rural:T30-Tapinoma sessile:Urban:T25 | 4.3464 | -10.4204 | 19.1132 | 0.9998 |
| Temnothorax longispinosus:Rural:T30-Tapinoma sessile:Urban:T25 | 10.1723 | -5.7777 | 26.1223 | 0.6818 |
| **Aphaenogaster picea:Urban:T30-Tapinoma sessile:Urban:T25** | **29.4012** | **14.1501** | **44.6523** | **<0.0001** |
| Tapinoma sessile:Urban:T30-Tapinoma sessile:Urban:T25 | 0.0206 | -17.0306 | 17.0719 | 1.0000 |
| **Temnothorax longispinosus:Urban:T30-Tapinoma sessile:Urban:T25** | **21.5275** | **7.1166** | **35.9385** | **0.0001** |
| Aphaenogaster picea:Rural:T30-Temnothorax longispinosus:Urban:T25 | -1.1947 | -12.8430 | 10.4535 | 1.0000 |
| Tapinoma sessile:Rural:T30-Temnothorax longispinosus:Urban:T25 | -8.9323 | -21.5778 | 3.7133 | 0.5044 |
| Temnothorax longispinosus:Rural:T30-Temnothorax longispinosus:Urban:T25 | -3.1063 | -17.1154 | 10.9027 | 1.0000 |
| **Aphaenogaster picea:Urban:T30-Temnothorax longispinosus:Urban:T25** | **16.1226** | **2.9147** | **29.3304** | **0.0040** |
| Tapinoma sessile:Urban:T30-Temnothorax longispinosus:Urban:T25 | -13.2580 | -28.5092 | 1.9931 | 0.1680 |
| Temnothorax longispinosus:Urban:T30-Temnothorax longispinosus:Urban:T25 | 8.2489 | -3.9792 | 20.4770 | 0.5873 |
| Tapinoma sessile:Rural:T30-Aphaenogaster picea:Rural:T30 | -7.7375 | -18.7441 | 3.2690 | 0.5131 |
| Temnothorax longispinosus:Rural:T30-Aphaenogaster picea:Rural:T30 | -1.9116 | -14.4610 | 10.6378 | 1.0000 |
| **Aphaenogaster picea:Urban:T30-Aphaenogaster picea:Rural:T30** | **17.3173** | **5.6691** | **28.9655** | **0.0001** |
| Tapinoma sessile:Urban:T30-Aphaenogaster picea:Rural:T30 | -12.0633 | -25.9856 | 1.8590 | 0.1720 |
| Temnothorax longispinosus:Urban:T30-Aphaenogaster picea:Rural:T30 | 9.4436 | -1.0807 | 19.9679 | 0.1325 |
| Temnothorax longispinosus:Rural:T30-Tapinoma sessile:Rural:T30 | 5.8259 | -7.6543 | 19.3061 | 0.9829 |
| **Aphaenogaster picea:Urban:T30-Tapinoma sessile:Rural:T30** | **25.0548** | **12.4093** | **37.7004** | **<0.0001** |
| Tapinoma sessile:Urban:T30-Tapinoma sessile:Rural:T30 | -4.3258 | -19.0926 | 10.4411 | 0.9998 |
| **Temnothorax longispinosus:Urban:T30-Tapinoma sessile:Rural:T30** | **17.1811** | **5.5626** | **28.7996** | **0.0001** |
| **Aphaenogaster picea:Urban:T30-Temnothorax longispinosus:Rural:T30** | **19.2289** | **5.2198** | **33.2379** | **0.0005** |
| Tapinoma sessile:Urban:T30-Temnothorax longispinosus:Rural:T30 | -10.1517 | -26.1017 | 5.7983 | 0.6850 |
| Temnothorax longispinosus:Urban:T30-Temnothorax longispinosus:Rural:T30 | 11.3552 | -1.7342 | 24.4446 | 0.1705 |
| **Tapinoma sessile:Urban:T30-Aphaenogaster picea:Urban:T30** | **-29.3806** | **-44.6317** | **-14.1295** | **<0.0001** |
| Temnothorax longispinosus:Urban:T30-Aphaenogaster picea:Urban:T30 | -7.8737 | -20.1018 | 4.3544 | 0.6664 |
| **Temnothorax longispinosus:Urban:T30-Tapinoma sessile:Urban:T30** | **21.5069** | **7.0959** | **35.9179** | **0.0001** |

| **Supplementary Table 9:** Post-hoc tests examining the differences in Defensive Behaviors between different Species. | | | | | |
| --- | --- | --- | --- | --- | --- |
| Contrast | estimate | SE | df | t.ratio | p.value |
| **Aphaenogaster picea - Tapinoma sessile** | **0.77** | **0.228** | **129** | **3.372** | **0.0028** |
| **Aphaenogaster picea - Temnothorax longispinosus** | **0.938** | **0.208** | **131** | **4.521** | **<0.0001** |
| Tapinoma sessile - Temnothorax longispinosus | 0.168 | 0.248 | 130 | 0.679 | 0.7764 |

| **Supplementary Table 10:** Post-hoc tests examining the differences in Defensive Behaviors between Rural and Urban Habitats. | | | | | |
| --- | --- | --- | --- | --- | --- |
| Contrast | estimate | SE | df | t.ratio | p.value |
| **Rural - Urban** | **-0.689** | **0.187** | **130** | **-3.689** | **0.0003** |

| **Supplementary Table 11:** Post-hoc tests examining the differences in Defensive Behaviors between different combinations of Species and Urbanization Status. | | | | | |
| --- | --- | --- | --- | --- | --- |
| Contrast | estimate | SE | df | t.ratio | p.value |
| Aphaenogaster picea Rural - Tapinoma sessile Rural | 0.2277 | 0.297 | 129 | 0.766 | 0.9727 |
| Aphaenogaster picea Rural - Temnothorax longispinosus Rural | 0.818 | 0.297 | 133 | 2.75 | 0.0723 |
| **Aphaenogaster picea Rural - Aphaenogaster picea Urban** | **-1.1302** | **0.26** | **129** | **-4.354** | **0.0004** |
| Aphaenogaster picea Rural - Tapinoma sessile Urban | 0.1821 | 0.323 | 129 | 0.564 | 0.9932 |
| Aphaenogaster picea Rural - Temnothorax longispinosus Urban | -0.0716 | 0.261 | 129 | -0.275 | 0.9998 |
| Tapinoma sessile Rural - Temnothorax longispinosus Rural | 0.5903 | 0.354 | 132 | 1.667 | 0.5557 |
| **Tapinoma sessile Rural - Aphaenogaster picea Urban** | **-1.3579** | **0.323** | **129** | **-4.206** | **0.0007** |
| Tapinoma sessile Rural - Tapinoma sessile Urban | -0.0456 | 0.376 | 129 | -0.121 | 1 |
| Tapinoma sessile Rural - Temnothorax longispinosus Urban | -0.2993 | 0.324 | 129 | -0.925 | 0.9395 |
| **Temnothorax longispinosus Rural - Aphaenogaster picea Urban** | **-1.9483** | **0.323** | **132** | **-6.031** | **<0.0001** |
| Temnothorax longispinosus Rural - Tapinoma sessile Urban | -0.636 | 0.376 | 131 | -1.692 | 0.5397 |
| Temnothorax longispinosus Rural - Temnothorax longispinosus Urban | -0.8897 | 0.324 | 132 | -2.747 | 0.0729 |
| **Aphaenogaster picea Urban - Tapinoma sessile Urban** | **1.3123** | **0.347** | **128** | **3.785** | **0.0032** |
| **Aphaenogaster picea Urban - Temnothorax longispinosus Urban** | **1.0586** | **0.289** | **128** | **3.657** | **0.0049** |
| Tapinoma sessile Urban - Temnothorax longispinosus Urban | -0.2537 | 0.347 | 128 | -0.73 | 0.9779 |

| **Supplementary Table 12:** Post-hoc tests examining the differences in Defensive Behaviors between different combinations of Species and Incubation Temperatures. | | | | | |
| --- | --- | --- | --- | --- | --- |
| Contrast | estimate | SE | df | t.ratio | p.value |
| Aphaenogaster picea T20 - Tapinoma sessile T20 | 0.6546 | 0.408 | 129 | 1.606 | 0.8001 |
| Aphaenogaster picea T20 - Temnothorax longispinosus T20 | 0.7196 | 0.361 | 136 | 1.992 | 0.5515 |
| Aphaenogaster picea T20 - Aphaenogaster picea T25 | -0.4932 | 0.32 | 129 | -1.54 | 0.8346 |
| Aphaenogaster picea T20 - Tapinoma sessile T25 | 1.1407 | 0.408 | 129 | 2.798 | 0.1261 |
| Aphaenogaster picea T20 - Temnothorax longispinosus T25 | 0.8087 | 0.375 | 129 | 2.158 | 0.4394 |
| Aphaenogaster picea T20 - Aphaenogaster picea T30 | 0.3613 | 0.32 | 129 | 1.128 | 0.969 |
| Aphaenogaster picea T20 - Tapinoma sessile T30 | 0.3828 | 0.382 | 130 | 1.001 | 0.9852 |
| **Aphaenogaster picea T20 - Temnothorax longispinosus T30** | **1.1546** | **0.355** | **129** | **3.253** | **0.0379** |
| Tapinoma sessile T20 - Temnothorax longispinosus T20 | 0.065 | 0.435 | 133 | 0.149 | 1 |
| Tapinoma sessile T20 - Aphaenogaster picea T25 | -1.1478 | 0.402 | 128 | -2.856 | 0.1097 |
| Tapinoma sessile T20 - Tapinoma sessile T25 | 0.4861 | 0.474 | 128 | 1.025 | 0.9828 |
| Tapinoma sessile T20 - Temnothorax longispinosus T25 | 0.1542 | 0.446 | 128 | 0.345 | 1 |
| Tapinoma sessile T20 - Aphaenogaster picea T30 | -0.2933 | 0.402 | 128 | -0.73 | 0.9983 |
| Tapinoma sessile T20 - Tapinoma sessile T30 | -0.2718 | 0.453 | 129 | -0.6 | 0.9996 |
| Tapinoma sessile T20 - Temnothorax longispinosus T30 | 0.5 | 0.43 | 128 | 1.163 | 0.9627 |
| **Temnothorax longispinosus T20 - Aphaenogaster picea T25** | **-1.2129** | **0.355** | **136** | **-3.419** | **0.0228** |
| Temnothorax longispinosus T20 - Tapinoma sessile T25 | 0.4211 | 0.435 | 133 | 0.968 | 0.9881 |
| Temnothorax longispinosus T20 - Temnothorax longispinosus T25 | 0.0891 | 0.405 | 134 | 0.22 | 1 |
| Temnothorax longispinosus T20 - Aphaenogaster picea T30 | -0.3584 | 0.355 | 136 | -1.01 | 0.9843 |
| Temnothorax longispinosus T20 - Tapinoma sessile T30 | -0.3369 | 0.412 | 135 | -0.818 | 0.9961 |
| Temnothorax longispinosus T20 - Temnothorax longispinosus T30 | 0.435 | 0.386 | 135 | 1.126 | 0.9693 |
| **Aphaenogaster picea T25 - Tapinoma sessile T25** | **1.6339** | **0.402** | **128** | **4.065** | **0.0026** |
| **Aphaenogaster picea T25 - Temnothorax longispinosus T25** | **1.302** | **0.369** | **128** | **3.533** | **0.0162** |
| Aphaenogaster picea T25 - Aphaenogaster picea T30 | 0.8545 | 0.313 | 128 | 2.73 | 0.1479 |
| Aphaenogaster picea T25 - Tapinoma sessile T30 | 0.876 | 0.376 | 129 | 2.328 | 0.3336 |
| **Aphaenogaster picea T25 - Temnothorax longispinosus T30** | **1.6478** | **0.348** | **128** | **4.731** | **0.0002** |
| Tapinoma sessile T25 - Temnothorax longispinosus T25 | -0.3319 | 0.446 | 128 | -0.743 | 0.998 |
| Tapinoma sessile T25 - Aphaenogaster picea T30 | -0.7794 | 0.402 | 128 | -1.939 | 0.588 |
| Tapinoma sessile T25 - Tapinoma sessile T30 | -0.7579 | 0.453 | 129 | -1.673 | 0.7615 |
| Tapinoma sessile T25 - Temnothorax longispinosus T30 | 0.0139 | 0.43 | 128 | 0.032 | 1 |
| Temnothorax longispinosus T25 - Aphaenogaster picea T30 | -0.4475 | 0.369 | 128 | -1.214 | 0.952 |
| Temnothorax longispinosus T25 - Tapinoma sessile T30 | -0.426 | 0.424 | 129 | -1.006 | 0.9848 |
| Temnothorax longispinosus T25 - Temnothorax longispinosus T30 | 0.3458 | 0.399 | 128 | 0.867 | 0.9943 |
| Aphaenogaster picea T30 - Tapinoma sessile T30 | 0.0215 | 0.376 | 129 | 0.057 | 1 |
| Aphaenogaster picea T30 - Temnothorax longispinosus T30 | 0.7933 | 0.348 | 128 | 2.277 | 0.3635 |
| Tapinoma sessile T30 - Temnothorax longispinosus T30 | 0.7718 | 0.406 | 129 | 1.9 | 0.6149 |

| **Supplementary Table 13:** Post-hoc tests examining the differences in CTmax between species. | | | | | |
| --- | --- | --- | --- | --- | --- |
| Contrast | estimate | SE | df | t.ratio | p.value |
| **Aphaenogaster picea - Tapinoma sessile** | **-4.04** | **0.312** | **126** | **-12.964** | **<0.0001** |
| **Aphaenogaster picea - Temnothorax longispinosus** | **-1.11** | **0.283** | **128** | **-3.915** | **0.0004** |
| **Tapinoma sessile - Temnothorax longispinosus** | **2.93** | **0.339** | **127** | **8.662** | **<0.0001** |

| **Supplementary Table 14:** Post-hoc tests examining the differences in CTmax between urban and rural sites. | | | | | |
| --- | --- | --- | --- | --- | --- |
| Contrast | estimate | SE | df | t.ratio | p.value |
| **Rural - Urban** | **-0.885** | **0.255** | **127** | **-3.473** | **0.0007** |

| **Supplementary Table 15:** Post-hoc tests examining the differences in CTmax between Incubation Temperatures. | | | | | |
| --- | --- | --- | --- | --- | --- |
| Contrast | estimate | SE | df | t.ratio | p.value |
| T20 - T25 | -0.593 | 0.319 | 127 | -1.86 | 0.1547 |
| **T20 - T30** | **-0.865** | **0.308** | **128** | **-2.812** | **0.0156** |
| T25 - T30 | -0.272 | 0.31 | 126 | -0.878 | 0.6553 |

| **Supplementary Table 16:** Post-hoc tests examining the differences in CTmax between different Species by Urbanization combinations. | | | | | |
| --- | --- | --- | --- | --- | --- |
| Contrast | estimate | SE | df | t.ratio | p.value |
| **Aphaenogaster picea Rural - Tapinoma sessile Rural** | **-3.2252** | **0.406** | **126** | **-7.946** | **<0.0001** |
| Aphaenogaster picea Rural - Temnothorax longispinosus Rural | -0.575 | 0.406 | 130 | -1.416 | 0.7172 |
| Aphaenogaster picea Rural - Aphaenogaster picea Urban | 0.0165 | 0.355 | 126 | 0.047 | 1 |
| **Aphaenogaster picea Rural - Tapinoma sessile Urban** | **-4.8443** | **0.441** | **126** | **-10.982** | **<0.0001** |
| **Aphaenogaster picea Rural - Temnothorax longispinosus Urban** | **-1.6271** | **0.356** | **126** | **-4.572** | **0.0002** |
| **Tapinoma sessile Rural - Temnothorax longispinosus Rural** | **2.6502** | **0.483** | **129** | **5.484** | **<0.0001** |
| **Tapinoma sessile Rural - Aphaenogaster picea Urban** | **3.2417** | **0.441** | **126** | **7.352** | **<0.0001** |
| **Tapinoma sessile Rural - Tapinoma sessile Urban** | **-1.6191** | **0.513** | **126** | **-3.155** | **0.024** |
| **Tapinoma sessile Rural - Temnothorax longispinosus Urban** | **1.598** | **0.442** | **126** | **3.615** | **0.0057** |
| Temnothorax longispinosus Rural - Aphaenogaster picea Urban | 0.5915 | 0.441 | 129 | 1.341 | 0.7615 |
| **Temnothorax longispinosus Rural - Tapinoma sessile Urban** | **-4.2693** | **0.513** | **128** | **-8.318** | **<0.0001** |
| Temnothorax longispinosus Rural - Temnothorax longispinosus Urban | -1.0521 | 0.442 | 129 | -2.38 | 0.1711 |
| **Aphaenogaster picea Urban - Tapinoma sessile Urban** | **-4.8608** | **0.474** | **125** | **-10.265** | **<0.0001** |
| **Aphaenogaster picea Urban - Temnothorax longispinosus Urban** | **-1.6437** | **0.395** | **125** | **-4.158** | **0.0008** |
| **Tapinoma sessile Urban - Temnothorax longispinosus Urban** | **3.2171** | **0.475** | **125** | **6.779** | **<0.0001** |

| **Supplementary Table 17:** Post-hoc tests examining the differences in CTmax between different Species by Incubation combinations. | | | | | |
| --- | --- | --- | --- | --- | --- |
| Contrast | estimate | SE | df | t.ratio | p.value |
| **Aphaenogaster picea T20 - Tapinoma sessile T20** | **-2.692** | **0.557** | **126** | **-4.836** | **0.0001** |
| Aphaenogaster picea T20 - Temnothorax longispinosus T20 | 0.038 | 0.493 | 133 | 0.077 | 1 |
| Aphaenogaster picea T20 - Aphaenogaster picea T25 | 0.402 | 0.438 | 126 | 0.92 | 0.9915 |
| **Aphaenogaster picea T20 - Tapinoma sessile T25** | **-3.922** | **0.557** | **126** | **-7.044** | **<0.0001** |
| Aphaenogaster picea T20 - Temnothorax longispinosus T25 | -0.915 | 0.512 | 126 | -1.788 | 0.6897 |
| Aphaenogaster picea T20 - Aphaenogaster picea T30 | 0.637 | 0.438 | 126 | 1.456 | 0.8731 |
| **Aphaenogaster picea T20 - Tapinoma sessile T30** | **-4.475** | **0.522** | **127** | **-8.569** | **<0.0001** |
| Aphaenogaster picea T20 - Temnothorax longispinosus T30 | -1.411 | 0.485 | 126 | -2.911 | 0.0959 |
| **Tapinoma sessile T20 - Temnothorax longispinosus T20** | **2.73** | **0.594** | **130** | **4.597** | **0.0003** |
| **Tapinoma sessile T20 - Aphaenogaster picea T25** | **3.095** | **0.549** | **125** | **5.638** | **<0.0001** |
| Tapinoma sessile T20 - Tapinoma sessile T25 | -1.229 | 0.648 | 125 | -1.897 | 0.6169 |
| Tapinoma sessile T20 - Temnothorax longispinosus T25 | 1.777 | 0.61 | 125 | 2.915 | 0.0951 |
| **Tapinoma sessile T20 - Aphaenogaster picea T30** | **3.33** | **0.549** | **125** | **6.066** | **<0.0001** |
| Tapinoma sessile T20 - Tapinoma sessile T30 | -1.783 | 0.619 | 126 | -2.883 | 0.1029 |
| Tapinoma sessile T20 - Temnothorax longispinosus T30 | 1.281 | 0.587 | 125 | 2.182 | 0.4238 |
| Temnothorax longispinosus T20 - Aphaenogaster picea T25 | 0.364 | 0.484 | 133 | 0.753 | 0.9978 |
| **Temnothorax longispinosus T20 - Tapinoma sessile T25** | **-3.959** | **0.594** | **130** | **-6.666** | **<0.0001** |
| Temnothorax longispinosus T20 - Temnothorax longispinosus T25 | -0.953 | 0.552 | 131 | -1.726 | 0.7292 |
| Temnothorax longispinosus T20 - Aphaenogaster picea T30 | 0.599 | 0.484 | 133 | 1.238 | 0.9465 |
| **Temnothorax longispinosus T20 - Tapinoma sessile T30** | **-4.513** | **0.562** | **131** | **-8.033** | **<0.0001** |
| Temnothorax longispinosus T20 - Temnothorax longispinosus T30 | -1.449 | 0.527 | 131 | -2.749 | 0.1413 |
| **Aphaenogaster picea T25 - Tapinoma sessile T25** | **-4.324** | **0.549** | **125** | **-7.878** | **<0.0001** |
| Aphaenogaster picea T25 - Temnothorax longispinosus T25 | -1.318 | 0.503 | 125 | -2.618 | 0.1897 |
| Aphaenogaster picea T25 - Aphaenogaster picea T30 | 0.235 | 0.427 | 125 | 0.549 | 0.9998 |
| **Aphaenogaster picea T25 - Tapinoma sessile T30** | **-4.878** | **0.514** | **126** | **-9.492** | **<0.0001** |
| **Aphaenogaster picea T25 - Temnothorax longispinosus T30** | **-1.813** | **0.476** | **125** | **-3.812** | **0.0065** |
| **Tapinoma sessile T25 - Temnothorax longispinosus T25** | **3.006** | **0.61** | **125** | **4.93** | **0.0001** |
| **Tapinoma sessile T25 - Aphaenogaster picea T30** | **4.559** | **0.549** | **125** | **8.306** | **<0.0001** |
| Tapinoma sessile T25 - Tapinoma sessile T30 | -0.554 | 0.619 | 126 | -0.896 | 0.9929 |
| **Tapinoma sessile T25 - Temnothorax longispinosus T30** | **2.51** | **0.587** | **125** | **4.275** | **0.0012** |
| Temnothorax longispinosus T25 - Aphaenogaster picea T30 | 1.552 | 0.503 | 125 | 3.085 | 0.0609 |
| **Temnothorax longispinosus T25 - Tapinoma sessile T30** | **-3.56** | **0.578** | **126** | **-6.155** | **<0.0001** |
| Temnothorax longispinosus T25 - Temnothorax longispinosus T30 | -0.496 | 0.545 | 125 | -0.91 | 0.992 |
| **Aphaenogaster picea T30 - Tapinoma sessile T30** | **-5.113** | **0.514** | **126** | **-9.949** | **<0.0001** |
| **Aphaenogaster picea T30 - Temnothorax longispinosus T30** | **-2.048** | **0.476** | **125** | **-4.306** | **0.0011** |
| **Tapinoma sessile T30 - Temnothorax longispinosus T30** | **3.064** | **0.555** | **126** | **5.525** | **<0.0001** |

| **Supplementary Table 18:** Post-hoc tests examining the differences in CTmax between different Urbanization by Incubation combinations. | | | | | |
| --- | --- | --- | --- | --- | --- |
| Contrast | estimate | SE | df | t.ratio | p.value |
| **Rural T20 - Urban T20** | **-2.3998** | **0.449** | **129** | **-5.349** | **<0.0001** |
| **Rural T20 - Rural T25** | **-1.3674** | **0.448** | **129** | **-3.055** | **0.032** |
| **Rural T20 - Urban T25** | **-2.2191** | **0.459** | **129** | **-4.833** | **0.0001** |
| **Rural T20 - Rural T30** | **-2.3633** | **0.429** | **130** | **-5.511** | **<0.0001** |
| **Rural T20 - Urban T30** | **-1.7665** | **0.446** | **129** | **-3.962** | **0.0017** |
| Urban T20 - Rural T25 | 1.0324 | 0.443 | 125 | 2.33 | 0.19 |
| Urban T20 - Urban T25 | 0.1808 | 0.455 | 125 | 0.398 | 0.9987 |
| Urban T20 - Rural T30 | 0.0365 | 0.424 | 126 | 0.086 | 1 |
| Urban T20 - Urban T30 | 0.6333 | 0.441 | 125 | 1.435 | 0.7057 |
| Rural T25 - Urban T25 | -0.8517 | 0.454 | 125 | -1.877 | 0.421 |
| Rural T25 - Rural T30 | -0.9959 | 0.423 | 126 | -2.354 | 0.1807 |
| Rural T25 - Urban T30 | -0.3991 | 0.44 | 125 | -0.907 | 0.9442 |
| Urban T25 - Rural T30 | -0.1442 | 0.435 | 126 | -0.331 | 0.9995 |
| Urban T25 - Urban T30 | 0.4525 | 0.452 | 125 | 1.001 | 0.9167 |
| Rural T30 - Urban T30 | 0.5967 | 0.421 | 126 | 1.417 | 0.7167 |

| **Supplementary Table 19:** Summarized Mantel Test results for each phenotype for each species. | | | |
| --- | --- | --- | --- |
| Species | Phenotype | r | p-value |
| A. picea | Colonization Rate | -0.0671 | 0.6035 |
|  | Recruitment Rate | -0.0664 | 0.6087 |
|  | Defensive Index | -0.2816 | 0.8652 |
|  | CTmax | 0.1998 | 0.1864 |
| T. sessile | Colonization Rate | -0.5007 | 0.9857 |
|  | Recruitment Rate | -0.2805 | 0.6504 |
|  | Defensive Index | -0.0357 | 0.5267 |
|  | CTmax | -0.2906 | 0.8039 |
| T. longispinosus | Colonization Rate | -0.2109 | 0.6126 |
|  | Recruitment Rate | -0.1706 | 0.8101 |
|  | Defensive Index | -0.2333 | 0.6639 |
|  | CTmax | -0.0719 | 0.5509 |
